# Molecular Determinants of O’Nyong-Nyong Virus Infection in Mammalian Hosts and *Anopheles* Mosquitoes

**DOI:** 10.3390/biom16060904

**Published:** 2026-06-18

**Authors:** Zhiyuan Liu, Xia Li, Hanwen Hu, Shangyu Xiao, Jianli Tao, Jing Yang

**Affiliations:** 1 Cuiying Biomedical Research Center, The Second Hospital & Clinical Medical School, Lanzhou University, Lanzhou 730030, China; lzhiyuan2023@lzu.edu.cn; 2 School of Basic Medical Sciences, Lanzhou University, Lanzhou 730000, China; lxia2024@lzu.edu.cn (X.L.); huhw2024@lzu.edu.cn (H.H.);; 3 Department of Pathology, Boston Children’s Hospital and Harvard Medical School, Boston, MA 02115, USA; jianli.tao@childrens.harvard.edu

**Keywords:** O’nyong-nyong virus, alphavirus, host–virus interaction, antiviral immunity, *Anopheles* mosquitoes, vector specificity

## Abstract

O’nyong-nyong virus (ONNV) is a mosquito-borne alphavirus responsible for large-scale epidemics in sub-Saharan Africa. As the closest evolutionary relative of Chikungunya virus (CHIKV), ONNV shares substantial genetic similarity and overlapping clinical manifestations with CHIKV. Mechanistic understanding of ONNV infection has therefore largely been extrapolated from CHIKV rather than directly established. However, ONNV exhibits distinct biological features, including predominant transmission by *Anopheles* mosquitoes and a clinical presentation characterized by prominent lymphadenopathy with limited acute joint edema. These distinctions underscore the need for an integrated synthesis of experimentally validated determinants of ONNV infection. In this review, we summarize current evidence on molecular and immunological factors regulating ONNV infection in mammalian hosts and mosquito vectors. We first discuss species-specific viral clearance, host dependency factors, intrinsic antiviral restriction mechanisms, protective innate immunity, inflammatory pathology, and mechanism-informed therapeutic strategies in mammalian hosts. We then examine stage-specific immune regulation in *Anopheles* mosquitoes, emphasizing mechanisms that constrain viral replication while permitting persistent infection and transmission. Finally, we discuss nsP3-dependent vector specificity and the potential contribution of alternative mosquito species to ONNV ecology. Together, this review provides an integrated framework for understanding how host factors, immune responses, and vector-specific adaptations shape ONNV infection, pathogenesis, and transmission.

## 1. Introduction

**Figure 1:**
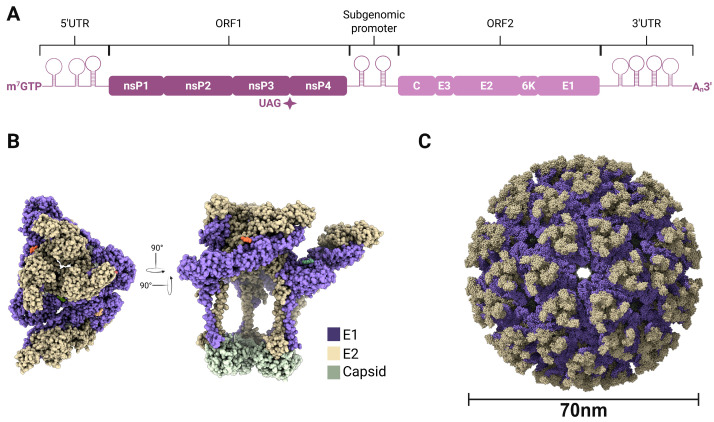
Genomic organization and virion structure of ONNV. (**A**) Schematic representation of the ONNV genome, showing the nonstructural proteins (nsP1–nsP4) and structural proteins (C, E3, E2, 6K, and E1). (**B**) Structural organization of the E1/E2 glycoprotein spike complex relative to the underlying capsid architecture. (**C**) Three-dimensional structure of a mature virion exhibiting icosahedral symmetry and an approximate diameter of 70 nm. Structural models were visualized in ChimeraX [13] based on the structure deposited in the Protein Data Bank (PDB ID: 6NK5). Figure created with BioRender.com by Z.L.

O’nyong-nyong virus (ONNV) belongs to the genus *Alphavirus* within the family *Togaviridae* and is serologically classified within the Semliki Forest virus complex [1,2]. This complex comprises several mosquito-borne alphaviruses, including Chikungunya virus (CHIKV), Ross River virus (RRV), Bebaru virus (BEBV), Semliki Forest virus (SFV), Mayaro virus (MAYV), and Una virus (UNAV). Notably, CHIKV, ONNV, RRV, and MAYV are classified as arthritogenic alphaviruses that cause joint-associated disease in humans [3,4]. As a member of this group, ONNV shares the canonical genomic architecture characteristic of alphaviruses. It possesses an approximately 11.8 kb single-stranded, capped, and polyadenylated positive-sense RNA genome organized into two open reading frames [5,6]. Following entry into host cells, the genomic RNA is translated to produce four nonstructural proteins (nsP1–4), which assemble into replication complexes responsible for the synthesis of a negative-strand RNA intermediate and the subsequent production of new genomic RNA and a subgenomic RNA. The subgenomic RNA directs translation of the structural polyprotein, which is processed into the capsid (C) protein and the envelope-associated proteins E3, E2, 6K, and E1 [7,8,9] (Figure 1A). Mature virions are enveloped, approximately 70 nm in diameter, and exhibit icosahedral symmetry. The capsid protein associates with the viral RNA to form the nucleocapsid, while E1 and E2 glycoproteins form heterodimers that are arranged into trimeric spikes on the viral envelope (Figure 1B). These structural proteins are assembled into a highly ordered virion architecture, with E1, E2, and capsid proteins generally present in approximately equimolar amounts in mature alphavirus particles (Figure 1C). In contrast, the nonstructural proteins function primarily during intracellular replication and are not components of the mature virion [10,11,12].

Since the massive 1959–1962 epidemic in Uganda that affected over two million individuals [14], ONNV has continued to circulate across multiple regions of Africa, with serological studies indicating substantial underrecognized transmission and sustained endemicity [15,16,17]. In natural transmission cycles, ONNV is maintained predominantly by malaria-transmitting *Anopheles* mosquitoes, especially *Anopheles gambiae* and *Anopheles funestus* [14,18,19,20,21,22,23,24]. This feature distinguishes ONNV from most other arthritogenic alphaviruses, which are usually transmitted by *Aedes* mosquitoes such as *Aedes aegypti* and *Aedes albopictus* [25,26]. Clinically, ONNV infection is generally self-limiting and characterized by fever, rash, pronounced myalgia, and debilitating polyarthralgia. Notably, compared with arthritogenic alphaviruses such as CHIKV and MAYV, ONNV-associated disease has been reported to show less prominent acute joint edema, while lymphadenopathy has been described as a distinctive clinical feature [22]. Phylogenetically, ONNV is closely related to CHIKV within the Semliki Forest virus complex [27]. Despite this close evolutionary relationship and substantial clinical overlap, its distinct pathological and ecological features indicate that high genetic conservation does not necessarily translate into identical host–virus interactions.

While previous reviews have provided thorough summaries of ONNV epidemiology, clinical features, and viral protein biology [28,29], a systematic integration of experimentally validated mammalian and mosquito determinants regulating ONNV infection remains to be established. Organized along the natural transmission cycle of ONNV, this review first examines infection from the perspective of the mammalian host. We discuss species-specific MARCO-mediated viral clearance as a host restriction mechanism that may help explain differences in viremia among susceptible and resistant hosts, highlighting key host-specific constraints on systemic viral dissemination. We then integrate host dependency factors, intrinsic antiviral restriction mechanisms, and immune-mediated inflammatory responses that collectively shape tissue tropism and disease outcome. Building on these insights, we synthesize mechanism-informed therapeutic strategies. The focus subsequently shifts to the mosquito vector. Antiviral immune responses in *Anopheles* mosquitoes are described from initial midgut infection to systemic dissemination, emphasizing mechanisms that limit viral replication, preserve vector fitness, and permit persistent infection and transmission. We further highlight nsP3 as a central determinant of ONNV’s selective compatibility with anopheline vectors and consider the potential contribution of additional mosquito species to ONNV transmission.

## 2. Species-Specific MARCO-Mediated Clearance Shapes ONNV Viremia and Host Susceptibility

Following mosquito-mediated dermal inoculation, ONNV is deposited into the cutaneous microenvironment, where local infection is expected to be initiated before systemic dissemination. Early target cell populations for ONNV remain incompletely defined and have often been inferred from studies of related arthritogenic alphaviruses, particularly CHIKV, in which keratinocytes, Langerhans cells, fibroblasts, and macrophages support productive infection and local amplification [3,30,31,32,33].

After initial replication at the site of inoculation, arboviruses disseminate through the lymphatic system, eventually gaining access to the systemic circulation [34,35]. Overcoming local immune containment within the draining lymph nodes (dLNs) is essential for the transition from localized replication to systemic dissemination, ultimately allowing the establishment of a viremia of sufficient magnitude and duration to support efficient mosquito acquisition. A critical determinant of whether systemic viremia is established lies in species-specific differences in the class A scavenger receptor MARCO (macrophage receptor with collagenous structure), which dictates the efficiency of intravascular alphavirus clearance and thereby shapes host susceptibility to sustained viremia. In mice, MARCO mediates rapid clearance of circulating ONNV and other arthritogenic alphaviruses, including CHIKV and RRV, with most virions eliminated from the bloodstream within 45 min. This process occurs through a sequential scavenger pathway. First, MARCO-expressing lymphatic endothelial cells in the draining lymph node capture lymph-borne virions independently of MXRA8 expression and CD169^+^ macrophages. Residual blood-borne virions are subsequently cleared by MARCO-expressing liver-resident macrophages, namely Kupffer cells. This process is independent of splenic function, complement activation, and antibody-mediated mechanisms [36,37]. MARCO-dependent viral uptake is mediated by its scavenger receptor cysteine-rich (SRCR) domain. Domain-swap and mutational analyses demonstrate that alphavirus internalization capacity is encoded within the SRCR domain itself rather than other receptor regions. Notably, replacing the murine SRCR domain with orthologous SRCR domains from different vertebrate species showed marked species-specific differences in viral uptake. SRCR domains from *Mus musculus*, *Bos taurus*, *Bubalus bubalis*, *Equus caballus*, and *Loxodonta africana* efficiently supported viral uptake. In contrast, SRCR domains from primates, including humans and rhesus macaques, as well as from rodent species other than *Mus musculus*, bats, and marsupials, did not support efficient uptake, despite similar receptor surface expression and preserved ligand-binding capacity. These findings indicate that subtle, species-specific divergence in SRCR surface chemistry directly dictates MARCO-mediated viral internalization. Such divergence may partly explain why humans, whose MARCO exhibits limited alphavirus internalization capacity, can develop viremia sufficient to support mosquito acquisition and thus serve as vertebrate hosts of ONNV [38]. Consistent with this mode of receptor-mediated recognition, MARCO-dependent clearance relies on conserved charged residues on the alphavirus envelope. In ONNV, mutational analysis confirms that residues corresponding to CHIKV E2-K200, E2-E208, and E1-K61 contribute to efficient clearance, consistent with CHIKV data, where mutation at these positions impairs clearance with limited tolerance for certain charge-preserving substitutions. In contrast, RRV depends on a more stringent and largely non-substitutable set of residues (e.g., E2-H232 and E2-D246), such that even conservative substitutions disrupt clearance [36,38].

## 3. Host Factors Underlying Selective Musculoskeletal and Stromal Tropism of ONNV

A murine infection study delineated the cellular tropism of ONNV, demonstrating predominant infection of CD45-non-immune stromal populations—including fibroblasts, endothelial cells, myoblasts, and mesenchymal stromal cells—whereas infiltrating leukocytes remained largely ONNV-negative [39]. The tissue and cellular tropism of ONNV may, at least in part, be attributable to cell-autonomous permissiveness shaped by the interplay between host factors exploited to support infection and host defense mechanisms that restrict infection across the viral life cycle.

**Figure 2:**
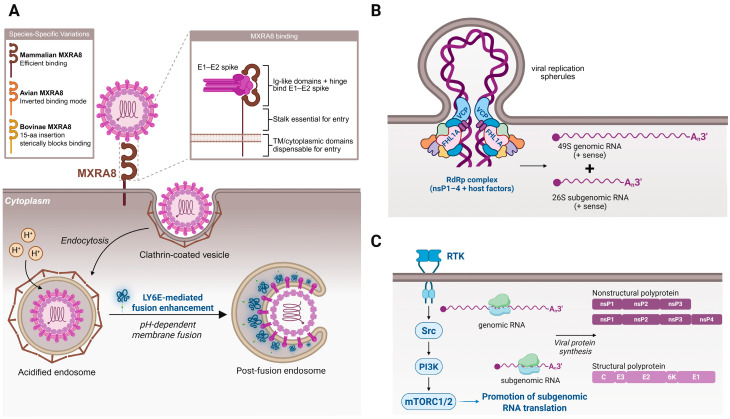
Host factors regulating ONNV entry, genome replication, and protein translation. (**A**) Entry. ONNV engages the receptor MXRA8, which facilitates viral attachment and entry, followed by low-pH-triggered membrane fusion. (**B**) Genome replication. Following nsP1–4 translation, replication complexes form to produce a negative-strand RNA intermediate and subsequently synthesize genomic and subgenomic RNAs. FHL1A, recruited by nsP3, promotes replication complex maturation and negative-strand synthesis, whereas VCP/p97 supports viral RNA synthesis at a post-entry stage. (**C**) Protein translation. Host receptor tyrosine kinase (RTK)–Src family kinase (SFK) signaling promotes PI3K–mTOR pathway activation, thereby enhancing subgenomic RNA translation and structural protein synthesis. Figure created with BioRender.com by Z.L.

At entry, ONNV engages MXRA8, a receptor shared among arthritogenic alphaviruses and expressed on keratinocytes, dermal and synovial fibroblasts, osteoblasts, chondrocytes, and skeletal muscle cells, consistent with the involvement of musculoskeletal and stromal tissues during infection [40]. In *Mxra8*^Δ8/Δ8^ mice expressing a truncated soluble receptor, ONNV titers in the ipsilateral ankle are reduced approximately sevenfold early after infection [41], underscoring the contribution of MXRA8 to early musculoskeletal infection. Structural studies have demonstrated that MXRA8 engages the viral E1–E2 spike within the inter-protomer “canyon” via its two Ig-like domains and hinge region, while its stalk region is indispensable for efficient viral entry [11,42]. Notably, comparative structural studies have shown that alphaviruses interact with MXRA8 in a host class-specific manner. Avian MXRA8 adopts an inverted binding mode compared with mammalian MXRA8 and preferentially supports infection by avian-reservoir alphaviruses [43]. In addition, species-specific structural variations can affect viral attachment. For example, a *Bovinae*-specific 15-amino-acid insertion in the receptor ectodomain sterically hinders viral binding and may contribute to species-level barriers to MXRA8-mediated entry [44]. Notably, MXRA8-mediated internalization does not require its transmembrane or cytoplasmic domains [45], suggesting that viral entry likely involves additional host factors. Given that classical receptor-mediated endocytosis typically depends on the coordinated action of adaptor proteins and clathrin-associated machinery, the molecular mechanisms by which virus–MXRA8 engagement triggers clathrin-mediated endocytosis remain unclear, and the specific host factors involved have yet to be defined. ONNV further exploits the interferon-inducible, glycosylphosphatidylinositol (GPI)-anchored surface protein LY6E to promote viral uncoating during entry, thereby enhancing infection in a cell type-dependent manner, particularly in fibroblast and monocytic lineages, without inducing broad transcriptional remodeling [46] (Figure 2A).

Following nonstructural protein translation and replication complex formation, ONNV replication is further modulated by host determinants. FHL1 functions as a highly specific host dependency factor for ONNV and CHIKV, with only the *FHL1A* splice variant supporting productive infection. Genetic ablation of FHL1 profoundly impairs negative-strand RNA synthesis, double-stranded RNA accumulation, replication organelle biogenesis, and infectious virion production, without affecting cellular viability. Conversely, ectopic expression of FHL1A converts otherwise non-permissive cells into a permissive state for infection. Mechanistically, ONNV nsP3 directly recruits FHL1 through a conserved motif (HVD R4) within its hypervariable domain, thereby promoting FHL1 incorporation into viral replication foci and facilitating efficient assembly and functional maturation of replication complexes. The high endogenous expression of FHL1 in skeletal and cardiac muscle is consistent with its role in supporting ONNV replication in musculoskeletal tissues. This model is supported by several findings. Cells derived from patients with FHL1-associated Emery–Dreifuss muscular dystrophy show reduced viral replication. In addition, *Fhl1*-deficient mice are protected from viral replication and myositis. These mice also show markedly reduced ONNV-induced viremia, musculoskeletal viral burden, inflammation, and joint pathology in vivo [47,48]. The conserved AAA^+^ ATPase VCP/p97 similarly promotes ONNV replication at a post-entry stage by supporting viral RNA synthesis in physiologically relevant human cell types, including fibroblasts and muscle cells. VCP inhibition reduces replication-dependent reporter activity without altering nonstructural protein abundance or RNA template availability, and VCP partially co-localizes with nsP1–3 within replication complexes [49] (Figure 2B).

At the level of structural protein synthesis, Src family kinases (SFKs) promote efficient translation of subgenomic RNAs through activation of phosphoinositide 3-kinase (PI3K)–protein kinase B (Akt)–mechanistic target of rapamycin (mTOR) signaling. SFK inhibition selectively reduces structural protein accumulation while sparing non-structural protein expression and redistributes subgenomic RNAs to monosomal fractions, decreasing translational efficiency by approximately 80% in a strictly replication-coupled manner [50]. The specific downstream effector proteins that execute this translational control, however, remain to be identified (Figure 2C).

## 4. Protective Immune Responses in ONNV Infection

**Figure 3:**
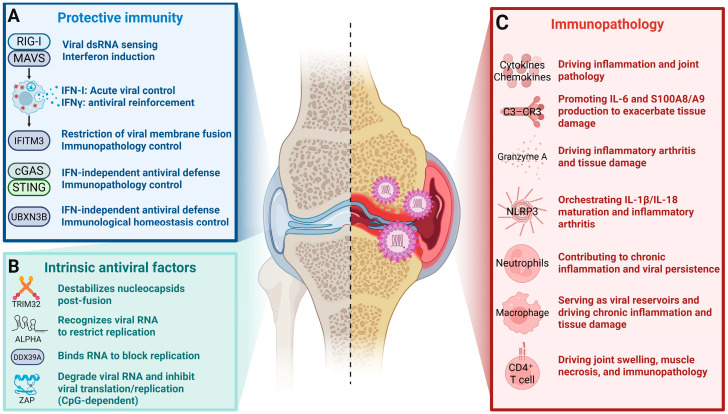
Protective versus Pathogenic Immunity and Mechanism-Informed Therapeutic Strategies in ONNV Infection. (**A**) Protective immunity. Early innate sensing pathways, including the RIG-I–MAVS axis, initiate IFN-I responses that mediate acute viral control, while IFN-γ reinforces antiviral restriction in specific biological contexts. Interferon-stimulated genes, such as IFITM3, limit pH-dependent membrane fusion and modulate viral dissemination and inflammatory amplification. Non-canonical STING-dependent pathways may further contribute to antiviral defense and inflammatory regulation during arthritogenic alphavirus infection. UBXN3B acts as an innate regulatory adaptor that coordinates antiviral restriction with the maintenance of immunological homeostasis. (**B**) Intrinsic antiviral factors. TRIM32 (post-fusion) destabilizes nucleocapsids and limits primary RNA translation; DDX39A (post-entry) restricts replication partly through recognition of conserved alphavirus RNA elements; ALPHA (lncRNA; RNA replication) suppresses antigenome synthesis by binding viral RNA within the nsp1 coding region; ZAP (replication, translation) inhibits early viral RNA translation in a CpG-dependent manner. (**C**) Immunopathology. Arthritogenic alphavirus infection can induce inflammatory arthritis and tissue damage through multiple inflammatory pathways. Cytokines, chemokines, complement signaling, granzyme A, and NLRP3 inflammasome activation have been implicated in disease progression in related alphavirus models. Inflammatory cell infiltration further amplifies pathology. Macrophages and neutrophils can contribute to chronic inflammation and viral persistence, whereas CD4^+^ T cells have been shown to drive ONNV-associated joint swelling and muscle necrosis. Figure created with BioRender.com by Z.L.

As viral RNA accumulates in infected cells, cytosolic and endosomal pattern-recognition receptors (PRRs), including retinoic acid-inducible gene I (RIG-I), melanoma differentiation-associated gene 5 (MDA5), and Toll-like receptor 7 (TLR7), become activated. RIG-I and MDA5 signal through MAVS-dependent pathways, whereas TLR7 activates MyD88-dependent signaling. Together, these pathways induce a robust type I interferon (IFN-I)-mediated antiviral response [51,52,53]. This early IFN-I response constitutes the dominant axis of host restriction during acute ONNV infection. Genetic ablation of IFN-I signaling leads to severe ONNV infection. A129 (*Ifnar1*^−/−^) mice and mice lacking the downstream effector Signal Transducer and Activator of Transcription 1 (STAT1) develop uncontrolled viremia, systemic viral dissemination, and lethal inflammatory disease. In contrast, wild-type mice, IFN-γ receptor-deficient mice, and RAG1-deficient mice show minimal viremia and no obvious clinical signs after infection with physiologically relevant mosquito-transmitted doses of the virus. These findings establish that innate IFN-I signaling is essential for acute systemic control and survival, while adaptive immunity and IFN-γ signaling are dispensable at the acute restriction stage [54]. Although dispensable for initial systemic control, IFN-γ contributes to antiviral defense in defined biological contexts. In immunocompetent mice, preexisting blood-stage infection with malaria parasites (e.g., *Plasmodium berghei* ANKA or *Plasmodium yoelii* 17XNL) suppresses ONNV-induced joint pathology and markedly reduces or abolishes viremia. Genetic deletion or in vivo neutralization of IFN-γ fully restores ONNV replication and dissemination under these conditions. *Plasmodium* infection confers durable resistance in early ONNV target cells, including fibroblasts, endothelial cells, myoblasts, and mesenchymal stromal cells, rather than simply delaying viral replication. Consistently, IFN-γ restricts ONNV infection in human fibroblast, synoviocyte, endothelial, and skeletal muscle cell lines, and plasma derived from *Plasmodium vivax*-infected patients suppresses ONNV infection in vitro in an IFN-γ receptor-dependent manner [39] (Figure 3A).

The antiviral state downstream of interferon signaling is executed by a broad repertoire of interferon-stimulated genes (ISGs). IFITM3 has emerged as an important ISG restricting alphavirus infection, including ONNV. IFITM3 does not impair viral attachment or internalization but inhibits pH-dependent membrane fusion, thereby limiting early cytosolic delivery of viral genomes. *Ifitm3*-deficient mice exhibit enhanced early viral dissemination, heightened inflammatory cytokine induction, and exacerbated joint pathology despite similar peak viral loads, demonstrating that early ISG-mediated containment shapes both viral kinetics and downstream inflammatory amplification [55]. Beyond IFITM3, antiviral defense against ONNV likely relies on a specific subset of ISGs [56], and systematic investigations are required to define the ISGs that mediate viral restriction and delineate their mechanisms (Figure 3A).

The canonical RIG-I–MAVS pathway represents a central axis for RNA virus recognition and control. In this pathway, cytosolic RIG-I detects viral RNA [57,58] and signal through the mitochondrial adaptor MAVS to activate IRF3 and NF-κB, thereby inducing type I interferons and antiviral gene expression [59,60,61,62]. Beyond this pathway, the cGAS–STING axis provides an additional layer of antiviral defense. The cGAS–STING pathway has been classically defined as a central cytosolic innate immune axis for the detection of DNA viruses [63,64,65,66]. Notably, accumulating evidence indicates that STING can also be engaged during RNA virus infection through non-canonical, DNA-independent mechanisms, expanding its role beyond classical DNA sensing. In the context of influenza A virus (IAV), STING is activated independently of canonical DNA sensing, and STING deficiency leads to increased viral titers both in vitro and in vivo, underscoring its antiviral function. Mechanistically, the Gly90 residue of STING is critical for recognizing the viral matrix protein M1 and for STING activation. Downstream of STING–NF-κB signaling, GADD34 functions as a key antiviral effector in the respiratory system [67]. During alphavirus infection, STING-dependent restriction also appears to operate through mechanisms that are not fully explained by canonical RNA sensing or classical IFN-I–STAT1 signaling. Pharmacological activation of STING suppresses ONNV and related alphaviruses by promoting type I interferon responses [68]. However, cGAS overexpression restricts ONNV replication even in STAT1-deficient fibroblasts [69]. The in vivo importance of STING signaling during arthritogenic alphavirus infection has been clearly demonstrated in CHIKV models. STING deficiency exacerbates CHIKV-induced arthritis and is accompanied by elevated viral burden in vivo. Notably, the expression of *Ifnα*, *Ifnβ*, and ISGs, including *Isg15*, *Oas1a*, *Ifit1* and *Ifit2*, is largely comparable between STING-deficient and wild-type mice. This indicates that the IFN-I response remains intact in the absence of STING during CHIKV infection, suggesting that STING restricts CHIKV infection through an interferon-independent mechanism [70]. Although these findings were established in the context of CHIKV infection, they provide important mechanistic insight into how STING-dependent pathways may contribute to antiviral defense and immune regulation during ONNV infection (Figure 3A).

The balance between antiviral restriction and inflammatory pathology is further shaped by regulatory adaptors that couple innate sensing to immune homeostasis. UBXN3B, which bridges STING to TRIM56 and promotes STING activation [71], exerts both cell-intrinsic antiviral and systemic immunoregulatory functions. *Ubxn3b*-deficient mice display enhanced early viremia and defective humoral immunity, characterized by reduced neutralizing antibody responses in the circulation. In parallel, exaggerated myeloid accumulation, reduced B and T cells, and elevated neutrophil-to-lymphocyte ratios are observed across the foot, spleen, and blood. Importantly, UBXN3B restricts alphavirus replication independently of intact IFN-I signaling. Loss of UBXN3B skews immune responses toward sustained proinflammatory cytokine production during the post-acute phase, illustrating how disruption of innate regulatory nodes can shift antiviral immunity toward prolonged inflammatory pathology [72] (Figure 3A).

## 5. Intrinsic Antiviral Factors Restricting ONNV Infection

In addition to the dominant interferon-driven antiviral program, host cells possess intrinsic antiviral mechanisms that function in a cell-autonomous manner and can act independently of, or in parallel with, IFN signaling. Early restriction of infection is mediated by the E3 ubiquitin ligase TRIM32. TRIM32 confers resistance to multiple alphaviruses, including ONNV, by localizing to endosome- and lysosome-associated compartments. It acts after viral fusion to destabilize incoming nucleocapsids and limit translation of primary genomic RNA, without affecting viral attachment or membrane fusion. However, the specific viral or host substrate targeted by TRIM32 has not yet been identified [73].

At a later stage of the viral life cycle, the DEAD-box helicase DDX39A acts as an interferon-independent restriction factor downstream of viral entry. Depletion of DDX39A enhances viral RNA accumulation and replication intermediate formation. This antiviral effect likely depends on the recognition of conserved alphavirus RNA structural elements, particularly within the 5′ untranslated region [74]. Complementing protein-based restriction mechanisms, the cytoplasmic long non-coding RNA ALPHA is expressed in human brain microvascular endothelial cells and is induced in a replication-dependent and alphavirus-specific manner. ALPHA directly binds viral genomic RNA within the nsP1 coding region through a minimal sequence encoded in exon 1, thereby suppressing early RNA replication, including antigenome synthesis, independently of IFN-I signaling [75]. This endothelial-intrinsic restriction mechanism may help explain the relatively limited neuroinvasion and weak neuroinflammatory responses observed in vivo following ONNV infection (Figure 3B).

The zinc finger antiviral protein (ZAP) exerts its broad-spectrum antiviral activity through multiple mechanisms, including viral RNA decay and direct inhibition of viral translation and replication [76,77,78]. Although its four alternative splice variants—ZAPS, ZAPM, ZAPL, and ZAPXL—differ in antiviral potency, with the longer isoforms generally exerting stronger restriction against alphaviruses, ONNV displays relatively attenuated sensitivity to ZAP-mediated restriction [78]. This resistance maps to the nonstructural gene region, particularly a discrete nsP2 segment in which localized CpG suppression limits effective ZAP restriction despite preserved RNA-binding capacity of both short and long isoforms, demonstrating that regional genomic adaptation rather than global CpG abundance determines the antiviral outcome [79].

Additional interferon-independent intrinsic modulators further shape cellular permissiveness: ectopic expression of SAMD4A, IRF1, P2RY6, HES4, MYD88, IRF2, or MAP3K14 confers robust restriction in STAT-deficient fibroblasts, whereas ADAR enhances viral replication [69]. The precise molecular mechanisms underlying these modulatory effects, however, remain to be elucidated. Collectively, these life cycle-stratified proviral and antiviral networks contribute to the selective musculoskeletal and stromal tropism of ONNV despite systemic hematogenous dissemination (Figure 3B).

## 6. Pathogenic Immune Responses in ONNV Infection

Although IFN-I-driven innate immunity effectively restricts systemic viral replication, localized musculoskeletal inflammation can become partially uncoupled from viral control and emerge as a major driver of disease pathogenesis. The clinical course of arthritogenic alphavirus infection is often biphasic. Acute disease typically emerges within several days after infection, temporally overlapping with early systemic viral replication and inflammatory cytokine induction [25,80,81]. It is characterized by elevated levels of IL-1β, IL-6, CXCL9, CCL2, CXCL10, and CCL5, many of which correlate with acute disease severity [82,83]. Notably, a substantial proportion of patients infected with arthritogenic alphaviruses, particularly CHIKV, fail to fully resolve musculoskeletal inflammation, progressing instead to a debilitating chronic rheumatic state characterized by persistent or relapsing arthralgia lasting months to years [84,85,86]. This progression is associated with temporally shifting cytokine networks, in which acute inflammatory mediators dominate early disease, whereas sustained IL-6 and GM-CSF signaling has been linked to chronic arthralgia [87].

Within the inflamed joint microenvironment, elevated chemokine signaling can promote inflammatory remodeling and tissue damage. For instance, increased levels of CCL2, CCL7, and CCL8 drive aberrant osteoclastogenesis and bone loss, which can be alleviated by bindarit, an inhibitor of CCL2, CCL7, and CCL8 production [88,89]. Similarly, macrophage migration inhibitory factor (MIF), a key pro-inflammatory mediator in rheumatoid arthritis [90], is markedly upregulated during RRV infection. Notably, MIF-deficient mice develop substantially attenuated disease, characterized by reduced inflammatory infiltration and muscle damage despite viral titers comparable to those of wild-type animals. Importantly, reconstitution of MIF in *MIF*^−/−^ mice restores severe disease manifestations, whereas pharmacological inhibition of MIF significantly ameliorates RRV-induced pathology in wild-type mice [91]. Acting in concert with these inflammatory networks, specific chemokine pathways further shape the course of CHIKV and ONNV infection, particularly the CXCL10 axis. Although CXCL10 is dispensable for systemic antiviral control, it critically regulates local tissue pathology. Loss or inhibition of CXCL10 does not affect early viral dissemination or viremia but accelerates viral clearance from infected joints and feet while attenuating local IFN-I and pro-inflammatory cytokine responses. This effect is accompanied by markedly reduced macrophage infiltration, whereas neutrophil and plasmacytoid dendritic cell recruitment remain largely CXCL10-independent. Importantly, infiltrating myeloid cells, particularly macrophages and neutrophils, can serve as cellular reservoirs for arthritogenic alphavirus replication in inflamed tissues, depending on the virus and disease stage. By promoting macrophage accumulation within infected tissues, CXCL10 signaling enhances local viral burden and thereby links chemokine-driven inflammation to sustained viral replication and immunopathology [92] (Figure 3C).

In parallel, the complement system contributes to alphavirus-induced immunopathology without significantly affecting viral replication or inflammatory cell infiltration. Instead, the C3–CR3 axis promotes tissue damage by enhancing the production of inflammatory mediators such as IL-6 and S100A8/A9 from infiltrating immune cells [93,94]. Similarly, cytotoxic effector molecules have been implicated in arthritogenic alphavirus-induced immunopathology. Functional studies demonstrated that granzyme A deficiency, and to a lesser extent granzyme K deficiency, markedly reduced CHIKV-induced foot swelling and arthritis, whereas granzyme B deficiency had minimal effects. Importantly, reduced disease severity in granzyme A-deficient mice occurred without changes in viral burden but was associated with diminished NK- and T-cell infiltration. Consistently, pharmacological inhibition of granzyme A using Serpinb6b also alleviated CHIKV-induced inflammatory arthritis in wild-type mice. Moreover, circulating granzyme A levels were elevated in both CHIKV-infected non-human primates and patients and correlated with viral load, supporting granzyme A as an important mediator of CHIKV immunopathology and a potential therapeutic target for CHIKV-associated arthritis [95].

Beyond soluble inflammatory mediators, chemokine networks, and cytotoxic effector molecules, intracellular innate immune sensing pathways also play critical roles in shaping alphavirus-induced immunopathology. Both human clinical samples and murine models of CHIKV [96] and MAYV [97] infection exhibit robust activation of the inflammasome pathway. Mechanistically, viral infection activates NLRP3 through ROS generation and potassium efflux, leading to caspase-1/11-dependent maturation of IL-1β and IL-18, whereas AIM2 appears largely dispensable [97]. Notably, disruption of the NLRP3 axis has minimal effects on viral burden in both CHIKV and MAYV infection models, suggesting that inflammasome signaling primarily regulates inflammatory pathology rather than systemic viral control [96,97]. In CHIKV infection, pharmacological inhibition of this pathway using the selective NLRP3 inhibitor MCC950 or the caspase-1 inhibitor Z-YVAD-FMK markedly attenuates joint inflammation and musculoskeletal pathology [96]. By contrast, genetic ablation of NLRP3 or caspase-1/11 during MAYV infection alters immune cell infiltration and moderately exacerbates foot swelling, indicating a more complex immunomodulatory role for inflammasome signaling in MAYV-associated disease [97]. Together, these findings identify the NLRP3 inflammasome as a key regulator of inflammatory balance during arthritogenic alphavirus infection and suggest that related inflammasome-dependent pathways may also be relevant to ONNV-associated inflammatory disease, although direct evidence in ONNV infection remains limited.

Together, these inflammatory pathways converge on coordinated cellular immune networks that shape the magnitude, localization, and persistence of musculoskeletal pathology. In RRV infection, disease progression is primarily orchestrated by a macrophage-dominated innate immune response. Macrophage depletion markedly attenuates arthritis and myositis while suppressing local production of pro-inflammatory mediators, including TNFα, CCL2, CCL3, IL-1β, and IFN-γ [98,99]. Consistent with this model, lymphocyte-deficient *Rag1^−/−^* mice develop musculoskeletal inflammation, macrophage/NK-cell infiltration, and tissue pathology comparable to those of wild-type animals [100], indicating that adaptive immune responses are largely dispensable for RRV-induced disease. Further supporting the pathogenic role of myeloid cells in RRV infection, arthritogenic alphavirus infection induces an arginase 1 (Arg1)-associated myeloid response involving macrophages and neutrophils within inflamed musculoskeletal tissues. Although typically associated with tissue repair, this response instead suppresses viral clearance and delays disease resolution. Consistently, myeloid-specific *Arg1* deficiency markedly reduces late-stage viral burden and ameliorates musculoskeletal pathology following RRV infection, implicating both macrophages and neutrophils in chronic inflammation and viral persistence [101]. By contrast, CHIKV-induced pathology relies more prominently on pathogenic adaptive immune responses. Studies using *Rag2^−/−^*, *Cd4^−/−^*, and *Cd8^−/−^* mice demonstrated that CD4^+^ T cells, rather than CD8^+^ T cells, are required for joint swelling and tissue injury, establishing CD4^+^ T-cell-driven inflammation as a central determinant of CHIKV musculoskeletal disease [102].

Similar CD4^+^ T cell-driven immunopathology has also been observed during ONNV infection. High-dimensional flow cytometry combined with longitudinal in vivo positron emission tomography (PET) imaging demonstrates pronounced accumulation of activated CD4^+^ T cells within infected joints at peak disease. Notably, ONNV-induced joint swelling and tissue injury occur in the absence of detectable vascular leakage or edema. This suggests that ONNV-associated joint pathology is driven primarily by immune-mediated tissue injury rather than overt vascular dysfunction. Histopathological analysis accordingly reveals prominent muscle degeneration, necrosis, and inflammatory cell infiltration. Functional dissection further uncouples viral control from disease pathogenesis: antibody-mediated CD4^+^ T cell depletion attenuates joint inflammation and muscle necrosis without altering viremia, whereas adoptive transfer of CD4^+^ T cells restores pathology in ONNV-infected TCR^−/−^ hosts [103] (Figure 3C).

Recent studies using single-cell RNA sequencing and spatial transcriptomics have further substantiated the pathogenic roles of CD4^+^ T cells and macrophages in arthritogenic alphavirus infection while providing important insights into the mechanisms underlying chronic disease persistence. During chronic infection with arthritogenic alphaviruses, including CHIKV, MAYV, and RRV, viral RNA persists in joint-associated tissues under immune control while sustaining low-level replication. Macrophages can serve as major viral reservoirs and adopt a pro-inflammatory MHC-II^high^ state, whereas infiltrating CD4^+^ T cells accumulate and produce IFN-γ. Spatial analyses further revealed close macrophage–CD4^+^ T cell interactions within inflamed tissues, suggesting that reciprocal immune activation sustains inflammatory circuits and thereby links persistent viral replication to chronic joint pathology [104]. Whether similar macrophage–CD4^+^ T cell spatial interactions contribute to persistent or chronic ONNV-associated musculoskeletal inflammation remains an important question for future investigation.

## 7. Mechanism-Informed Intervention Strategies

Mechanism-informed therapeutic strategies against ONNV can be organized along the sequential stages of infection and host response. The most immediate antiviral approach targets essential components of the viral replication machinery. The conserved alphavirus nsP2 protease represents a tractable catalytic vulnerability: the small-molecule inhibitor RA-00002034 potently suppresses replication of both virulent and attenuated ONNV strains in normal human dermal fibroblasts by disrupting viral polyprotein processing, demonstrating activity across distinct ONNV strains [105,106]. Numerous compounds targeting CHIKV viral proteins have demonstrated potent antiviral activity in experimental settings, and given the high degree of protein conservation between CHIKV and ONNV, these inhibitors may provide useful starting points for evaluating cross-reactive antiviral activity against ONNV [105,107,108,109,110] (Table 1). Among emerging viral RNA-dependent RNA polymerase (RdRp) inhibitors, VV261 has recently shown notable promise as a broad-spectrum antiviral candidate against CHIKV. Originally developed for severe fever with thrombocytopenia syndrome virus (SFTSV) and reported to be under Phase I clinical evaluation, VV261 overcomes the poor aqueous stability of its parent nucleoside analog [111]. In vitro, VV261 exhibits potent and dose-dependent antiviral activity against multiple CHIKV lineages, including the Asian and East/Central/South African (ECSA) genotypes, as well as several other alphaviruses. Mechanistically, VV261 acts during the post-entry stage of viral replication; as a pyrimidine analog, it competitively interferes with nascent viral RNA synthesis without inducing lethal mutagenesis. In A129 mouse models, low-dose oral administration of VV261 effectively suppresses viremia and reduces viral burdens in joint-associated tissues to undetectable levels. Importantly, this antiviral effect is accompanied by marked attenuation of footpad swelling and significant reductions in the local expression of pro-inflammatory cytokines and chemokines [107]. Although direct validation against ONNV remains important, these findings support viral polymerase inhibition as a mechanism-informed strategy with potential relevance to ONNV.

Beyond direct inhibition of viral replication machinery, viral entry constitutes a therapeutically tractable bottleneck centered on the interaction between the E2 glycoprotein and the host receptor MXRA8. Soluble MXRA8–Fc fusion proteins function as decoy receptors that competitively sequester virions away from endogenous cell-surface MXRA8 in vivo, thereby blocking viral attachment and entry [40]. Monoclonal antibodies directed against MXRA8 can similarly sterically occlude the receptor and prevent virus–receptor engagement [40]. In addition to receptor-targeted strategies, several broadly neutralizing antibodies originally generated against CHIKV, including CHK-187 and CHK-265, cross-neutralize ONNV by targeting conserved residues within the E2 B domain and confer in vivo protection against arthritis and dissemination [112]. The human monoclonal antibody RRV-12, isolated from infected donors, neutralizes virus entry by blocking the E2–MXRA8 interaction [113]. Owing to the high structural conservation within the Semliki Forest antigenic complex, the licensed CHIKV vaccine IXCHIQ (VLA1553) elicits cross-neutralizing antibodies that provide partial cross-protection against ONNV in experimental settings [114]. In contrast to epitope-centered strategies, host-factor-guided attenuation offers a mechanistically distinct paradigm: attenuated viruses such as CHIKV-ΔFHL1 can retain immunogenicity while losing pathogenicity, thereby conferring cross-protection against ONNV [48] (Table 1).

Beyond defined host–virus interfaces, broader host-directed pharmacologic strategies target intracellular signaling and metabolic networks that support viral replication. Inhibition of Src family kinases impairs structural protein translation [50], while activation of Rev-erb nuclear receptors suppresses late-stage viral protein accumulation and dampens inflammatory gene expression [115]. Plant-derived compounds such as berberine and tabersonine further illustrate the feasibility of dual antiviral–anti-inflammatory modulation [116]. Finally, amplification of innate immunity represents an upstream therapeutic strategy. Among innate immune agonists, STING agonists, including cAIMP, show potent antiviral activity by inducing IFN-I responses, suppressing alphavirus replication, and mitigating inflammatory arthritis in vivo [117]. Complementing direct antiviral approaches, post-infection treatment with fingolimod (FTY720), an FDA-approved sphingosine-1-phosphate receptor modulator, suppresses CD4^+^ T cell trafficking into the joints and reduces disease severity without impairing viral clearance. These findings further support the idea that ONNV-associated arthralgia can be driven substantially by dysregulated host immune responses rather than by uncontrolled viral replication alone [103] (Table 1).

## 8. Stage-Specific Immune Regulation of ONNV Infection in Anopheles Mosquitoes

Successful transmission of ONNV requires efficient establishment of infection within its *Anopheles* mosquito vectors, where viral replication must be balanced by innate immune responses in a manner that allows vector survival, persistent infection, and onward transmission [118]. Following ingestion of an infectious blood meal, ONNV establishes infection in the mosquito midgut, the primary anatomical and immunological checkpoint within the vector.

**Figure 4:**
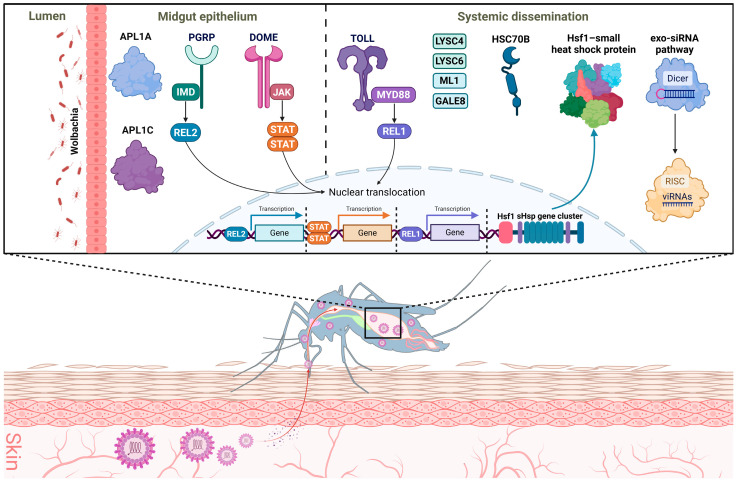
Compartment-specific antiviral factors restricting ONNV infection in *Anopheles* mosquitoes. In the midgut epithelium, antiviral signaling is primarily mediated by the Imd and JAK/STAT pathways. The leucine-rich repeat proteins APL1A and APL1C act as restrictive extracellular immune factors during oral infection, independently of the classical APL1C–LRIM1–TEP1 complement-like cascade. In addition, the midgut microbiota can promote ONNV establishment, whereas *Wolbachia*-mediated inhibition of ONNV has been demonstrated in experimental *Aedes*-derived systems and may inform vector-control strategies. Following breach of the midgut barrier and entry into the hemocoel, systemic control of ONNV is dominated by the exogenous small interfering RNA pathway. Additional extracellular modulators, including ML1, GALE8, and lysozyme-type effectors (LYSC4 and LYSC6), exert modulatory antiviral effects. Stress-responsive pathways also contribute to restriction, as Hsf1-driven induction of the clustered small heat shock protein genes and HSC70B activity limit viral replication during systemic infection. Factors depicted are those with experimental evidence for restriction or modulation of ONNV infection in mosquito systems. Figure created with BioRender.com by Z.L.

Leucine-rich repeat proteins, APL1A and APL1C, are classically characterized as extracellular immune factors associated with the complement-like defense system in *Anopheles* mosquitoes. In anti-*Plasmodium* defense, APL1C forms a functional complex with LRIM1 and TEP1 and is typically associated with TEP1-dependent parasite killing and melanization [119,120,121]. APL1A, in turn, is transcriptionally regulated by the Rel2-S isoform of the immune deficiency (Imd) pathway transcription factor [121]. During ONNV infection, however, this canonical regulatory organization appears to be functionally uncoupled. Silencing of *APL1A* or *APL1C* enhances ONNV titers following oral exposure, indicating that both factors impose restrictive pressure on viral infection. Depletion of *LRIM1*, *TEP1*, or the related *TEP3* does not alter ONNV infection, suggesting that this antiviral activity operates independently of the classical APL1C–LRIM1–TEP1 complement-like cascade [122]. At the signaling level, antiviral control in the midgut is primarily associated with the Imd and JAK/STAT pathways. Knockdown of the *Rel2-F* isoform, the transcriptional effector of the Imd pathway, markedly increases viral susceptibility, as does silencing of *Stat-A*, a central component of JAK/STAT signaling, indicating that midgut-restricted antiviral defense relies on Rel2-F- and Stat-A-dependent transcriptional programs. By contrast, depletion of the Toll pathway regulator *Rel1* produces no detectable in vivo effect during the midgut phase of infection. Likewise, although the exogenous small interfering RNA pathway is active in this tissue, knockdown of *Ago2* or *Dcr2* fails to alter viral loads at this early stage [122] (Figure 4).

The midgut microbiota overall appears to facilitate ONNV infection rather than acting as a purely antiviral barrier. Antibiotic-mediated depletion of resident gut bacteria markedly reduces viral titers following oral challenge, whereas reconstitution with live bacteria, but not heat-inactivated bacteria, restores infectivity, indicating that an intact and metabolically active microbial community promotes viral establishment within the midgut. However, this proviral effect does not imply that all bacterial taxa are beneficial to ONNV. Instead, the microbial community likely contains both virus-permissive and virus-restrictive members. Consistent with this view, dsRNA-mediated silencing of the antimicrobial peptide *CEC3* decreases viral titers during early infection, suggesting that CEC3 may influence ONNV infection indirectly through microbiota-dependent mechanisms, although a direct antiviral or immunomodulatory role cannot be excluded [122]. *Wolbachia* are intracellular endosymbiotic bacteria widely present in arthropods that can influence pathogen transmission by manipulating host reproduction and suppressing pathogen replication. Leveraging these properties, *Wolbachia*-based strategies represent a promising paradigm for the control of mosquito-borne diseases [123,124]. Although these studies were performed in *Aedes*-derived Aa23 cells and stably transinfected *Ae. aegypti* mosquitoes rather than natural *Anopheles* vectors, *Wolbachia* infection significantly reduces ONNV infection in these experimental systems. Mechanistically, *Wolbachia*-mediated ONNV inhibition appears to be independent of lipid droplet cholesterol storage, since treatment with 2-hydroxypropyl-β-cyclodextrin (2HPCD), a cholesterol-depleting agent, fails to rescue viral replication [125] (Figure 4).

After breaching the midgut barrier and initiating systemic dissemination, ONNV encounters a markedly altered immune landscape. During systemic infection established by intrathoracic inoculation, classical innate immune signaling pathways appear to play a limited role in controlling viral replication. Silencing of core components of the JAK/STAT pathway (HOP, STAT1/STAT2, and PIAS), the Toll pathway (REL1 and CACT), and the Imd pathway (REL2) has little to no impact on viral replication [126]. Mechanistic studies in hemocyte-like 4a3A cells provide a possible explanation for this compartment-dependent difference. ONNV broadly suppresses Toll, Imd, and JAK/STAT signaling pathways. Although activation of the Toll pathway alone can restrict viral replication, simultaneous activation of the JAK/STAT, Imd, and Toll pathways weakens and eventually abolishes this antiviral effect [122]. These findings suggest that immune network cross-talk may attenuate the antiviral output of classical signaling pathways during systemic infection. Nevertheless, extracellular immune components exert measurable modulatory effects. Depletion of pathogen recognition receptors (ML1, GALE8) or lysozyme-type effectors (LYSC4, LYSC6) augments viral replication, yet does not alter infection prevalence [126]. Instead, systemic ONNV control is dominated by the exogenous small interfering RNA (exo-siRNA) pathway. Indeed, the first description of siRNA as a mosquito antiviral mechanism emerged from studies of *An. gambiae* infected with ONNV [127], establishing RNA interference as a central antiviral defense in this vector. Viral double-stranded RNA is processed by Dicer-2 into ~21-nt virus-derived siRNAs that are incorporated into AgAgo2-containing RISC complexes to mediate sequence-specific degradation of viral RNA [128]. Functional analyses reveal a clear Argonaute hierarchy, with AgAgo2 serving as the principal antiviral effector: its depletion markedly enhances viral replication, systemic dissemination, tissue permissiveness, and mosquito mortality, whereas other Argonaute proteins contribute minimally [127]. In parallel, stress-responsive heat shock pathways constitute an additional layer of cell-intrinsic antiviral defense. Knockdown of the heat shock protein HSC70B elevates whole-body viral titers and enhances tissue-level dissemination, accompanied by reduced survival [129]. Complementing this, recent multi-omics analyses have identified a conserved Hsf1–small heat shock protein (sHsp) cascade that exerts antiviral activity at an early post-entry stage of viral RNA replication. Upon viral infection, Hsf1 coordinately induces eight sHsp genes clustered within a single genomic locus, reflecting tight cis-regulatory control. This pathway restricts diverse arboviruses across mosquito species, including CHIKV in *Aedes* cells and ONNV in *An. gambiae* Mos55 cells, and retains antiviral activity in RNAi-deficient contexts [130] (Figure 4).

## 9. ONNV nsP3 Governs Selective Compatibility with Anopheles Mosquitoes

Divergence of ONNV and CHIKV likely occurred only thousands of years ago [27], whereas their principal mosquito vectors, *An. gambiae* and *Ae. aegypti*, separated during the Jurassic period [131]. This temporal mismatch suggests that relatively limited viral genetic variation was sufficient to establish a pronounced vector restriction across deeply diverged mosquito lineages. Laboratory infection studies support this restriction: *An. gambiae* is highly susceptible to ONNV but refractory to CHIKV infection [132].

Chimeric virus analyses have delineated the viral determinants of mosquito specificity. Exchange of structural genes between ONNV and CHIKV showed that incorporation of ONNV structural proteins into a CHIKV backbone conferred only limited [133], and in some experimental settings no [134], competence for infection of *An. gambiae*, indicating that structural proteins alone do not determine *Anopheles* tropism. In contrast, substitution of nsP3 produced a markedly different outcome: only chimeras retaining intact ONNV nsP3 achieved infection efficiencies in *An. gambiae* comparable to parental ONNV, whereas replacement of ONNV nsP3 with its CHIKV counterpart abolished viral viability in both mammalian and insect cells [134]. Thus, ONNV nsP3 emerges as a major viral determinant underlying its capacity to infect *Anopheles* mosquitoes. Accordingly, comparative immunoprecipitation–proteomic analyses in Sua4.0 cells revealed divergent host interaction networks assembled by ONNV and CHIKV nsP3. ONNV nsP3 uniquely associates with WD repeat-containing protein 48 (WDR48), huntingtin-interacting protein (HIP1), cathepsin L, fermitin, breast cancer metastasis-suppressor 1-like protein, and 28S ribosomal protein S22, indicating the presence of an ONNV-specific host interaction module in anopheline cells [135]. Therefore, nsP3-dependent tropism likely reflects combinatorial engagement of multiple host factors. Whether these specialized interactions directly underlie ONNV tropism remains to be determined and will require targeted loss-of-function analyses in both cell lines and intact mosquitoes.

As a multifunctional component of the alphavirus RNA replicase, nsP3 exhibits a modular architecture comprising an N-terminal macrodomain, a central alphavirus-unique/zinc-binding domain (AUD/ZBD), and a C-terminal hypervariable domain (HVD). Through coordinated activities of these domains, nsP3 regulates replication complex assembly, modulates viral RNA synthesis, and mediates interactions with host factors [136,137,138,139,140,141,142,143,144]. Sequence divergence within nsP3 between ONNV and CHIKV thus provides a plausible molecular basis for their differential replication capacities in *Anopheles* cells. Dissecting how specific amino acid differences alter domain-specific functions and host interactions will be essential for defining the mechanistic basis of nsP3-dependent vector tropism.

An additional regulatory layer resides at the nsP3/nsP4 junction. The opal stop codon (UGA) between nsP3 and nsP4 governs the production of two polyproteins—P123 and P1234—through translational termination and readthrough, respectively [145]. Low-passage ONNV isolates retain the opal codon, which can convert to arginine (CGA) during serial passage in Vero cells, with mixed populations frequently detected, consistent with quasispecies dynamics [5]. Although the opal codon attenuates replication in vertebrate and mosquito cell lines, it confers a marked fitness advantage in the natural mosquito vector, enhancing transmission potential [146]. This finding indicates that regulated readthrough at the nsP3/nsP4 boundary represents a finely tuned mechanism balancing replication efficiency and transmission fitness across vertebrate and arthropod hosts.

The recent establishment of a CRISPR drop-out screening system in an anopheline cell line Sua-5B provides a powerful framework for systematic identification of genes essential for anopheline cellular function [147]. Although this platform was not originally developed to study ONNV infection, it provides a methodological foundation for future genetic screens in anopheline cells. Application of chimeric reporter viruses or ONNV-based infection models in CRISPR-based genetic screens could enable unbiased identification of anopheline host factors required for ONNV replication or nsP3-dependent compatibility, thereby facilitating functional validation and mechanistic characterization of the effector pathways that govern ONNV tropism.

## 10. Potential Expansion of ONNV Beyond Anopheles Gambiae and Anopheles Funestus

ONNV is currently regarded as geographically confined to sub-Saharan Africa [29], where transmission has been sustained primarily by *An. gambiae* and *An. Funestus* [14,18,19,20,21,22,23,24]. This spatial restriction has often been interpreted as evidence of biological dependence on a relatively narrow vector spectrum. Yet such a view risks conflating ecological circumstance with intrinsic constraint. Rather than reflecting a fixed virological boundary, ONNV’s distribution may represent a historically contingent ecological equilibrium—one structured by vector availability, environmental suitability, and patterns of mammalian host–mosquito contact.

That equilibrium may become increasingly unstable. Intensifying globalization, climate change, and the redistribution of invasive mosquito species are reshaping vector ecology. With nearly one quarter of recognized human disease vectors now documented beyond their historical native ranges [148], geographic barriers that once constrained arboviral transmission are progressively weakening. Under these conditions, ONNV’s apparent confinement should not be assumed to represent permanent stability. As vector ranges expand and human mobility accelerates, cross-regional dissemination becomes a plausible epidemiological scenario rather than merely theoretical.

The identification of an imported ONNV infection in Germany in 2013 following travel to Kenya [149] provides evidence that human mobility can introduce ONNV beyond endemic regions. Although no secondary transmission was detected—indicating that local ecological conditions were insufficient for sustained spread at that time—the absence of observed transmission does not necessarily imply inherent containment. Arboviral emergence is conditional: when viral introduction coincides with competent mosquito vectors and susceptible mammalian hosts, localized importations may transition into sustained transmission under favorable ecological conditions.

The global expansion of Zika virus offers a contemporary illustration of this dynamic. Following introduction into regions where competent *Aedes* vectors were already widely established, Zika shifted rapidly from sporadic importations to epidemic spread in immunologically naïve populations, producing substantial public health and socio-economic consequences [150,151]. By analogy, ONNV’s present geographic restriction cannot be assumed to guarantee future containment. Systematic identification and characterization of additional mosquito species with potential competence for ONNV therefore represents an important preparedness priority, aimed at anticipating ecological opportunities for emergence rather than responding only after transmission becomes established.

Of particular concern is the rapid expansion of *Anopheles stephensi*, an invasive Asian species within the broader anopheline vector landscape. Now established across the Horn of Africa and spreading southward [152], *An. stephensi* is a highly adaptable urban malaria vector capable of transmitting both *Plasmodium falciparum* and *P. vivax* [153]. Experimental studies demonstrate efficient ONNV infection following infectious blood feeding, with viral RNA detected in salivary glands [154]—a key indicator of potential transmission competence. Similarly, *Anopheles coluzzii* is a major Afrotropical malaria vector within the *An. gambiae* complex, distributed across western and central sub-Saharan Africa and broadly overlapping with *An. gambiae* across diverse ecological settings. Despite its relatively recent divergence, it exhibits distinct larval ecology, exploiting both transient rain-fed pools and anthropogenic permanent habitats, and shows marked ecological plasticity, including flexible host choice and indoor–outdoor biting and resting behavior, as well as persistence through dry seasons [155]. Importantly, it has been experimentally demonstrated to support infection with ONNV under laboratory conditions [156], and its ecological overlap with established vectors suggests a potential role in ONNV transmission under favorable circumstances. Beyond Africa, *Anopheles albimanus*, a predominant anopheline species distributed throughout Central and South America, also exhibits high laboratory competence for ONNV, while remaining refractory to CHIKV and flaviviruses [157]. Susceptibility further extends beyond anophelines. The globally invasive mosquito *Ae. albopictus* has been shown to acquire, replicate, and transmit ONNV under permissive temperature conditions, whereas native European *Culex* species remain refractory [158]. These laboratory competence data indicate that ONNV vector range may be broader than historically recognized, although field relevance will depend on vector abundance, feeding behavior, ecological overlap with vertebrate hosts, and environmental suitability.

## 11. Conclusions

As the closest known relative of CHIKV, ONNV shares substantial genetic and biological overlap with CHIKV, but its distinctive vector usage, clinical presentation, and host–vector interactions indicate that it should not be regarded simply as an ecological variant of CHIKV. Current evidence instead supports viewing ONNV as a biologically distinct arthritogenic alphavirus whose infection is shaped by an integrated network of mammalian host determinants, antiviral restriction mechanisms, immune-mediated pathology, and vector-specific viral adaptations. In mammalian hosts, species-specific MARCO-mediated clearance may influence systemic viremia, whereas MXRA8, LY6E, FHL1, VCP/p97, Src family kinases, and multiple intrinsic antiviral factors regulate viral entry, replication, tissue tropism, and cellular permissiveness. At the immune level, IFN-I signaling provides a dominant axis of acute viral restriction, while inflammatory pathways involving chemokines, complement, inflammasome activation, macrophages, and CD4^+^ T cells can drive musculoskeletal pathology that is partially uncoupled from systemic viral control. These mechanisms also reveal potential intervention points, including viral replication machinery, receptor-mediated entry, host dependency factors, innate immune activation, and immunopathology-directed therapies.

In mosquito vectors, ONNV infection is shaped by stage-specific immune regulation. Midgut-associated Imd and JAK–STAT responses, microbiota-dependent effects, systemic exo-siRNA activity, and stress-responsive heat shock pathways collectively influence viral replication, dissemination, persistence, and vector fitness. Among viral determinants, nsP3 has emerged as a major driver of ONNV compatibility with anopheline mosquitoes, although the host factors and interaction networks that execute nsP3-dependent vector tropism remain incompletely defined. Laboratory susceptibility of additional *Anopheles* species and *Ae. albopictus* suggests that ONNV vector range may be broader than historically recognized, but the field relevance of these observations will depend on vector ecology, feeding behavior, environmental suitability, and opportunities for human–mosquito contact.

Future studies should therefore prioritize ONNV-specific experimental systems, functional validation of host and vector determinants, and integrated analysis of how viral replication, immune regulation, tissue pathology, and mosquito compatibility jointly shape ONNV biology. Together, these efforts will clarify why ONNV shares important features with CHIKV yet follows a distinct mechanistic, pathogenic, and ecological trajectory.

## Figures and Tables

**Table 1:** Mechanism-informed intervention strategies with relevance to ONNV infection and disease.

Intervention	Mechanism or Rationale	References
RA-00002034	Targets the conserved nsP2 protease to block viral polyprotein processing and replication	[105,106]
VV261	Broad-spectrum RdRp inhibitor; suppresses alphavirus RNA synthesis during post-entry replication with potential relevance to ONNV	[107,111]
Soluble MXRA8–Fc	Acts as a decoy receptor; competitively sequesters free virions away from endogenous cell-surface MXRA8, blocking viral attachment and entry	[40]
Anti-MXRA8 mAbs	Induces steric occlusion of the host cell-surface receptor, physically blocking virus–receptor engagement	[40]
Broadly Neutralizing mAbs (e.g., CHK-187, CHK-265)	Target conserved residues within the E2 B domain of CHIKV; cross-neutralize ONNV to protect against arthritis and viral dissemination in vivo	[112]
RRV-12 mAb	Human-derived mAb that prevents viral entry by specifically disrupting the E2–MXRA8 interaction	[113]
IXCHIQ (VLA1553) (Licensed CHIKV Vaccine)	Exploits high structural conservation within the Semliki Forest antigenic complex to elicit cross-neutralizing antibodies, conferring partial protection against ONNV	[114]
CHIKV-ΔFHL1	Host-factor-guided attenuation paradigm; abolishes pathogenicity while preserving robust immunogenicity to confer cross-protection against ONNV	[48]
Src Family Kinase Inhibitors	Perturb host intracellular signaling networks to selectively impair viral structural protein translation	[50]
Rev-erb Nuclear Receptor Agonists	Suppress late-stage viral protein accumulation and simultaneously dampen host pro-inflammatory gene expression	[115]
Plant-derived Compounds (e.g., Berberine, Tabersonine)	Target host metabolic and signaling cascades to achieve a dual antiviral and anti-inflammatory modulatory effect in alphavirus infection models	[116]
STING Agonists (e.g., cAIMP)	Upstream innate immune agonists; trigger potent IFN-I responses to broadly restrict ONNV and related alphaviruses and mitigate inflammatory arthritis in vivo	[117]
Fingolimod (FTY720)	Immunopathology control strategy; post-infection treatment blocks CD4^+^ T-cell trafficking into inflamed joints and alleviates arthritis without impairing viral clearance	[103]

## Data Availability

No new data were created or analyzed in this study. Data sharing is not applicable to this article.

## Abbreviations

The following abbreviations are used in this manuscript:

ALPHA	Antiviral lncRNA prohibiting human alphaviruses
APL1A/APL1C	*Anopheles Plasmodium*-responsive leucine-rich repeat protein 1A/1C
CHIKV	Chikungunya Virus
CXCL10	C-X-C Motif Chemokine Ligand 10
DDX39A	DEAD-Box Helicase 39A
dLNs	Draining Lymph Nodes
FHL1	Four and a Half LIM Domains Protein 1
IFN-I	Type I Interferon
IFN-γ	Interferon Gamma
IFITM3	Interferon-Induced Transmembrane Protein 3
ISGs	Interferon-Stimulated Genes
JAK/STAT	Janus Kinase/Signal Transducer and Activator of Transcription
LY6E	Lymphocyte Antigen 6 Family Member E
MARCO	Macrophage Receptor with Collagenous Structure
MAVS	Mitochondrial Antiviral Signaling Protein
MDA5	Melanoma Differentiation-Associated Protein 5
MXRA8	Matrix Remodeling Associated Protein 8
NF-κB	Nuclear Factor Kappa B
nsP1–4	Nonstructural Proteins 1–4
ONNV	O’nyong-nyong Virus
PBMCs	Peripheral Blood Mononuclear Cells
PI3K–Akt–mTOR	Phosphoinositide 3-Kinase–Protein Kinase B–Mammalian Target of Rapamycin
PRRs	Pattern Recognition Receptors
RIG-I	Retinoic Acid-Inducible Gene I
RNAi	RNA Interference
RRV	Ross River Virus
SFK	Src Family Kinases
siRNA	Small Interfering RNA
STING	STimulator of INterferon Genes
TLR7	Toll-Like Receptor 7
TRIM32	Tripartite Motif Containing 32
UBXN3B	UBX Domain Protein 3B
VCP/p97	Valosin-Containing Protein
ZAP	Zinc Finger Antiviral Protein

## References

[1] Chen R., Mukhopadhyay S., Merits A., Bolling B., Nasar F., Coffey L.L., Powers A., Weaver S.C., Ictv Report C. (2018). ICTV Virus Taxonomy Profile: Togaviridae. J. Gen. Virol..

[2] Williams M.C., Woodall J.P., Porterfield J.S. (1962). O’nyong-nyong fever: An epidemic virus disease in East Africa: V. Human antibody studies by plaque inhibition and other serological tests. Trans. R. Soc. Trop. Med. Hyg..

[3] Kafai N.M., Diamond M.S., Fox J.M. (2022). Distinct Cellular Tropism and Immune Responses to Alphavirus Infection. Annu. Rev. Immunol..

[4] Raju S., Adams L.J., Diamond M.S. (2024). The many ways in which alphaviruses bind to cells. Trends Immunol..

[5] Lanciotti R.S., Ludwig M.L., Rwaguma E.B., Lutwama J.J., Kram T.M., Karabatsos N., Cropp B.C., Miller B.R. (1998). Emergence of epidemic O’nyong-nyong fever in Uganda after a 35-year absence: Genetic characterization of the virus. Virology.

[6] Ledermann J.P., Kayiwa J.T., Perinet L.C., Apangu T., Acayo S., Lutwama J.J., Powers A.M., Mossel E.C. (2022). Complete Genome Sequence of O’nyong Nyong Virus Isolated from a Febrile Patient in 2017 in Uganda. Microbiol. Resour. Announc..

[7] Rupp J.C., Sokoloski K.J., Gebhart N.N., Hardy R.W. (2015). Alphavirus RNA synthesis and non-structural protein functions. J. Gen. Virol..

[8] Kääriäinen L., Ahola T. (2002). Functions of alphavirus nonstructural proteins in RNA replication. Prog. Nucleic Acid. Res. Mol. Biol..

[9] Leung J.Y., Ng M.M., Chu J.J. (2011). Replication of alphaviruses: A review on the entry process of alphaviruses into cells. Adv. Virol..

[10] Yap M.L., Klose T., Urakami A., Hasan S.S., Akahata W., Rossmann M.G. (2017). Structural studies of Chikungunya virus maturation. Proc. Natl. Acad. Sci. USA.

[11] Basore K., Kim A.S., Nelson C.A., Zhang R., Smith B.K., Uranga C., Vang L., Cheng M., Gross M.L., Smith J. (2019). Cryo-EM Structure of Chikungunya Virus in Complex with the Mxra8 Receptor. Cell.

[12] Button J.M., Qazi S.A., Wang J.C.-Y., Mukhopadhyay S. (2020). Revisiting an old friend: New findings in alphavirus structure and assembly. Curr. Opin. Virol..

[13] Goddard T.D., Huang C.C., Meng E.C., Pettersen E.F., Couch G.S., Morris J.H., Ferrin T.E. (2018). UCSF ChimeraX: Meeting modern challenges in visualization and analysis. Protein Sci..

[14] Haddow A.J., Davies C.W., Walker A.J. (1960). O’nyong-nyong fever: An epidemic virus disease in East Africa 1. Introduction. Trans. R. Soc. Trop. Med. Hyg..

[15] Bowen E.T., Simpson D.I., Platt G.S., Way H., Bright W.F., Day J., Achapa S., Roberts J.M. (1973). Large scale irrigation and arbovirus epidemiology, Kano Plain, Kenya. II. Preliminary serological survey. Trans. R. Soc. Trop. Med. Hyg..

[16] Hozé N., Diarra I., Sangaré A.K., Pastorino B., Pezzi L., Kouriba B., Sagara I., Dabo A., Djimdé A., Thera M.A. (2021). Model-based assessment of Chikungunya and O’nyong-nyong virus circulation in Mali in a serological cross-reactivity context. Nat. Commun..

[17] Masika M.M., Korhonen E.M., Smura T., Uusitalo R., Ogola J., Mwaengo D., Jääskeläinen A.J., Alburkat H., Gwon Y.-D., Evander M. (2022). Serological Evidence of Exposure to Onyong-Nyong and Chikungunya Viruses in Febrile Patients of Rural Taita-Taveta County and Urban Kibera Informal Settlement in Nairobi, Kenya. Viruses.

[18] Williams M.C., Woodall J.P., Corbet P.S., Gillett J.D. (1965). O’nyong-Nyong Fever: An Epidemic Virus Disease in East Africa. 8. Virus Isolations from *Anopheles* Mosquitoes. Trans. R. Soc. Trop. Med. Hyg..

[19] Johnson B.K., Gichogo A., Gitau G., Patel N., Ademba G., Kirui R., Highton R.B., Smith D.H. (1981). Recovery of o’nyong-nyong virus from *Anopheles funestus* in Western Kenya. Trans. R. Soc. Trop. Med. Hyg..

[20] Corbet P.S., Williams M.C., Gillett J.D. (1961). O’Nyong-Nyong fever: An epidemic virus disease in East Africa. IV. Vector studies at epidemic sites. Trans. R. Soc. Trop. Med. Hyg..

[21] Williams M.C., Woodall J.P. (1961). O’nyong-nyong fever: An epidemic virus disease in East Africa. II. Isolation and some properties of the virus. Trans. R. Soc. Trop. Med. Hyg..

[22] Kiwanuka N., Sanders E.J., Rwaguma E.B., Kawamata J., Ssengooba F.P., Najjemba R., Were W.A., Lamunu M., Bagambisa G., Burkot T.R. (1999). O’nyong-nyong fever in south-central Uganda, 1996–1997: Clinical features and validation of a clinical case definition for surveillance purposes. Clin. Infect. Dis..

[23] Posey D.L., O’Rourke T., Roehrig J.T., Lanciotti R.S., Weinberg M., Maloney S. (2005). O’Nyong-nyong fever in West Africa. Am. J. Trop. Med. Hyg..

[24] Pezzi L., LaBeaud A.D., Reusken C.B., Drexler J.F., Vasilakis N., Diallo M., Simon F., Jaenisch T., Gallian P., Sall A. (2019). GloPID-R report on chikungunya, o’nyong-nyong and Mayaro virus, part 2: Epidemiological distribution of o’nyong-nyong virus. Antivir. Res..

[25] de Souza W.M., Lecuit M., Weaver S.C. (2025). Chikungunya virus and other emerging arthritogenic alphaviruses. Nat. Rev. Microbiol..

[26] Zaid A., Burt F.J., Liu X., Poo Y.S., Zandi K., Suhrbier A., Weaver S.C., Texeira M.M., Mahalingam S. (2021). Arthritogenic alphaviruses: Epidemiological and clinical perspective on emerging arboviruses. Lancet Infect. Dis..

[27] Powers A.M., Brault A.C., Tesh R.B., Weaver S.C. (2000). Re-emergence of Chikungunya and O’nyong-nyong viruses: Evidence for distinct geographical lineages and distant evolutionary relationships. J. Gen. Virol..

[28] Rezza G., Chen R., Weaver S.C. (2017). O’nyong-nyong fever: A neglected mosquito-borne viral disease. Pathog. Glob. Health.

[29] Tong Jia Ming S., Tan Yi Jun K., Carissimo G. (2024). Pathogenicity and virulence of O’nyong-nyong virus: A less studied Togaviridae with pandemic potential. Virulence.

[30] Young A.R., Locke M.C., Cook L.E., Hiller B.E., Zhang R., Hedberg M.L., Monte K.J., Veis D.J., Diamond M.S., Lenschow D.J. (2019). Dermal and muscle fibroblasts and skeletal myofibers survive chikungunya virus infection and harbor persistent RNA. PLoS Pathog..

[31] Felipe V.L.J., Paula A.V., Silvio U.I. (2020). Chikungunya virus infection induces differential inflammatory and antiviral responses in human monocytes and monocyte-derived macrophages. Acta Trop..

[32] Sourisseau M., Schilte C., Casartelli N., Trouillet C., Guivel-Benhassine F., Rudnicka D., Sol-Foulon N., Le Roux K., Prevost M.C., Fsihi H. (2007). Characterization of reemerging chikungunya virus. PLoS Pathog..

[33] Lentscher A.J., McCarthy M.K., May N.A., Davenport B.J., Montgomery S.A., Raghunathan K., McAllister N., Silva L.A., Morrison T.E., Dermody T.S. (2019). Chikungunya virus replication in skeletal muscle cells is required for disease development. J. Clin. Investig..

[34] MacDonald G.H., Johnston R.E. (2000). Role of dendritic cell targeting in Venezuelan equine encephalitis virus pathogenesis. J. Virol..

[35] Johnston L.J., King N.J.C., Halliday G.M. (2000). Langerhans Cells Migrate to Local Lymph Nodes Following Cutaneous Infection with an Arbovirus. J. Investig. Dermatol..

[36] Carpentier K.S., Davenport B.J., Haist K.C., McCarthy M.K., May N.A., Robison A., Ruckert C., Ebel G.D., Morrison T.E. (2019). Discrete viral E2 lysine residues and scavenger receptor MARCO are required for clearance of circulating alphaviruses. eLife.

[37] Carpentier K.S., Sheridan R.M., Lucas C.J., Davenport B.J., Li F.S., Lucas E.D., McCarthy M.K., Reynoso G.V., May N.A., Tamburini B.A.J. (2021). MARCO(+) lymphatic endothelial cells sequester arthritogenic alphaviruses to limit viremia and viral dissemination. Embo J..

[38] Li F.S., Carpentier K.S., Hawman D.W., Lucas C.J., Ander S.E., Feldmann H., Morrison T.E. (2023). Species-specific MARCO-alphavirus interactions dictate chikungunya virus viremia. Cell Rep..

[39] Torres-Ruesta A., Teo T.H., Chan Y.H., Amrun S.N., Yeo N.K., Lee C.Y., Nguee S.Y., Tay M.Z., Nosten F., Fong S.W. (2022). Malaria abrogates O’nyong-nyong virus pathologies by restricting virus infection in nonimmune cells. Life Sci. Alliance.

[40] Zhang R., Kim A.S., Fox J.M., Nair S., Basore K., Klimstra W.B., Rimkunas R., Fong R.H., Lin H., Poddar S. (2018). Mxra8 is a receptor for multiple arthritogenic alphaviruses. Nature.

[41] Zhang R., Earnest J.T., Kim A.S., Winkler E.S., Desai P., Adams L.J., Hu G., Bullock C., Gold B., Cherry S. (2019). Expression of the Mxra8 Receptor Promotes Alphavirus Infection and Pathogenesis in Mice and Drosophila. Cell Rep..

[42] Song H., Zhao Z., Chai Y., Jin X., Li C., Yuan F., Liu S., Gao Z., Wang H., Song J. (2019). Molecular Basis of Arthritogenic Alphavirus Receptor MXRA8 Binding to Chikungunya Virus Envelope Protein. Cell.

[43] Zimmerman O., Zimmerman M.I., Raju S., Nelson C.A., Errico J.M., Madden E.A., Holmes A.C., Hassan A.O., VanBlargan L.A., Kim A.S. (2023). Vertebrate-class-specific binding modes of the alphavirus receptor MXRA8. Cell.

[44] Kim A.S., Zimmerman O., Fox J.M., Nelson C.A., Basore K., Zhang R., Durnell L., Desai C., Bullock C., Deem S.L. (2020). An Evolutionary Insertion in the Mxra8 Receptor-Binding Site Confers Resistance to Alphavirus Infection and Pathogenesis. Cell Host Microbe.

[45] Feng F., Bouma E.M., Hu G., Zhu Y., Yu Y., Smit J.M., Diamond M.S., Zhang R. (2023). Colocalization of Chikungunya Virus with Its Receptor MXRA8 during Cell Attachment, Internalization, and Membrane Fusion. J. Virol..

[46] Mar K.B., Rinkenberger N.R., Boys I.N., Eitson J.L., McDougal M.B., Richardson R.B., Schoggins J.W. (2018). LY6E mediates an evolutionarily conserved enhancement of virus infection by targeting a late entry step. Nat. Commun..

[47] Meertens L., Hafirassou M.L., Couderc T., Bonnet-Madin L., Kril V., Kümmerer B.M., Labeau A., Brugier A., Simon-Loriere E., Burlaud-Gaillard J. (2019). FHL1 is a major host factor for chikungunya virus infection. Nature.

[48] Ng W.H., Liu X., Ling Z.L., Santos C.N.O., Magalhães L.S., Kueh A.J., Herold M.J., Taylor A., Freitas J.R., Koit S. (2023). FHL1 promotes chikungunya and o’nyong-nyong virus infection and pathogenesis with implications for alphavirus vaccine design. Nat. Commun..

[49] Carissimo G., Chan Y.-H., Utt A., Chua T.-K., Bakar F.A., Merits A., Ng L.F.P. (2019). VCP/p97 Is a Proviral Host Factor for Replication of Chikungunya Virus and Other Alphaviruses. Front. Microbiol..

[50] Broeckel R., Sarkar S., May N.A., Totonchy J., Kreklywich C.N., Smith P., Graves L., DeFilippis V.R., Heise M.T., Morrison T.E. (2019). Src Family Kinase Inhibitors Block Translation of Alphavirus Subgenomic mRNAs. Antimicrob. Agents Chemother..

[51] Rehwinkel J., Gack M.U. (2020). RIG-I-like receptors: Their regulation and roles in RNA sensing. Nat. Rev. Immunol..

[52] Hartmann G. (2017). Nucleic Acid Immunity. Adv. Immunol..

[53] Goubau D., Deddouche S., Reis e Sousa C. (2013). Cytosolic sensing of viruses. Immunity.

[54] Seymour R.L., Rossi S.L., Bergren N.A., Plante K.S., Weaver S.C. (2013). The role of innate versus adaptive immune responses in a mouse model of O’nyong-nyong virus infection. Am. J. Trop. Med. Hyg..

[55] Poddar S., Hyde J.L., Gorman M.J., Farzan M., Diamond M.S. (2016). The Interferon-Stimulated Gene IFITM3 Restricts Infection and Pathogenesis of Arthritogenic and Encephalitic Alphaviruses. J. Virol..

[56] McDougal M.B., De Maria A.M., Ohlson M.B., Kumar A., Xing C., Schoggins J.W. (2023). Interferon inhibits a model RNA virus via a limited set of inducible effector genes. EMBO Rep..

[57] Yoneyama M., Kikuchi M., Natsukawa T., Shinobu N., Imaizumi T., Miyagishi M., Taira K., Akira S., Fujita T. (2004). The RNA helicase RIG-I has an essential function in double-stranded RNA-induced innate antiviral responses. Nat. Immunol..

[58] Kato H., Takeuchi O., Sato S., Yoneyama M., Yamamoto M., Matsui K., Uematsu S., Jung A., Kawai T., Ishii K.J. (2006). Differential roles of MDA5 and RIG-I helicases in the recognition of RNA viruses. Nature.

[59] Xu L.G., Wang Y.Y., Han K.J., Li L.Y., Zhai Z., Shu H.B. (2005). VISA is an adapter protein required for virus-triggered IFN-beta signaling. Mol. Cell.

[60] Seth R.B., Sun L., Ea C.K., Chen Z.J. (2005). Identification and characterization of MAVS, a mitochondrial antiviral signaling protein that activates NF-kappaB and IRF 3. Cell.

[61] Meylan E., Curran J., Hofmann K., Moradpour D., Binder M., Bartenschlager R., Tschopp J. (2005). Cardif is an adaptor protein in the RIG-I antiviral pathway and is targeted by hepatitis C virus. Nature.

[62] Kawai T., Takahashi K., Sato S., Coban C., Kumar H., Kato H., Ishii K.J., Takeuchi O., Akira S. (2005). IPS-1, an adaptor triggering RIG-I- and Mda5-mediated type I interferon induction. Nat. Immunol..

[63] Wu J., Sun L., Chen X., Du F., Shi H., Chen C., Chen Z.J. (2013). Cyclic GMP-AMP Is an Endogenous Second Messenger in Innate Immune Signaling by Cytosolic DNA. Science.

[64] Ishikawa H., Barber G.N. (2008). STING is an endoplasmic reticulum adaptor that facilitates innate immune signalling. Nature.

[65] Zhong B., Yang Y., Li S., Wang Y.-Y., Li Y., Diao F., Lei C., He X., Zhang L., Tien P. (2008). The Adaptor Protein MITA Links Virus-Sensing Receptors to IRF3 Transcription Factor Activation. Immunity.

[66] Sun W., Li Y., Chen L., Chen H., You F., Zhou X., Zhou Y., Zhai Z., Chen D., Jiang Z. (2009). ERIS, an endoplasmic reticulum IFN stimulator, activates innate immune signaling through dimerization. Proc. Natl. Acad. Sci. USA.

[67] Ye R., Wang S., Hu Y., Pan Y., Zheng W., Xia F., Wang Y., Guo H., Zheng S., Wei W. (2026). STING–NF-κB signaling builds an influenza spillover barrier. Science.

[68] Gall B., Pryke K., Abraham J., Mizuno N., Botto S., Sali T.M., Broeckel R., Haese N., Nilsen A., Placzek A. (2018). Emerging Alphaviruses Are Sensitive to Cellular States Induced by a Novel Small-Molecule Agonist of the STING Pathway. J. Virol..

[69] Schoggins J.W., MacDuff D.A., Imanaka N., Gainey M.D., Shrestha B., Eitson J.L., Mar K.B., Richardson R.B., Ratushny A.V., Litvak V. (2014). Pan-viral specificity of IFN-induced genes reveals new roles for cGAS in innate immunity. Nature.

[70] Geng T., Lin T., Yang D., Harrison A.G., Vella A.T., Fikrig E., Wang P. (2020). A Critical Role for STING Signaling in Limiting Pathogenesis of Chikungunya Virus. J. Infect. Dis..

[71] Yang L., Wang L., Ketkar H., Ma J., Yang G., Cui S., Geng T., Mordue D.G., Fujimoto T., Cheng G. (2018). UBXN3B positively regulates STING-mediated antiviral immune responses. Nat. Commun..

[72] Geng T., Yang D., Lin T., Cahoon J.G., Wang P. (2022). UBXN3B Controls Immunopathogenesis of Arthritogenic Alphaviruses by Maintaining Hematopoietic Homeostasis. mBio.

[73] Xie Y., Cao J., Gan S., Xu L., Zhang D., Qian S., Xu F., Ding Q., Schoggins J.W., Fan W. (2024). TRIM32 inhibits Venezuelan equine encephalitis virus infection by targeting a late step in viral entry. PLoS Pathog..

[74] Tapescu I., Taschuk F., Pokharel S.M., Zginnyk O., Ferretti M., Bailer P.F., Whig K., Madden E.A., Heise M.T., Schultz D.C. (2023). The RNA helicase DDX39A binds a conserved structure in chikungunya virus RNA to control infection. Mol. Cell.

[75] Basavappa M.G., Ferretti M., Dittmar M., Stoute J., Sullivan M.C., Whig K., Shen H., Liu K.F., Schultz D.C., Beiting D.P. (2022). The lncRNA *ALPHA* specifically targets chikungunya virus to control infection. Mol. Cell.

[76] Shao R., Xi C., Zhou D., Guan J., Huang Y., Qi Y., Liu X., van Oers M., Fros J.J., Yin X. (2026). ZAP inhibits double-stranded RNA virus infection by degrading negative-strand RNA and blocking the elongation phase of viral protein synthesis. Cell Rep..

[77] Bick M.J., Carroll J.-W.N., Gao G., Goff S.P., Rice C.M., MacDonald M.R. (2003). Expression of the Zinc-Finger Antiviral Protein Inhibits Alphavirus Replication. J. Virol..

[78] Li M.M.H., Aguilar E.G., Michailidis E., Pabon J., Park P., Wu X., de Jong Y.P., Schneider W.M., Molina H., Rice C.M. (2019). Characterization of Novel Splice Variants of Zinc Finger Antiviral Protein (ZAP). J. Virol..

[79] Nguyen L.P., Aldana K.S., Yang E., Yao Z., Li M.M.H. (2023). Alphavirus Evasion of Zinc Finger Antiviral Protein (ZAP) Correlates with CpG Suppression in a Specific Viral nsP2 Gene Sequence. Viruses.

[80] Silva L.A., Dermody T.S. (2017). Chikungunya virus: Epidemiology, replication, disease mechanisms, and prospective intervention strategies. J. Clin. Investig..

[81] Simon F., Javelle E., Oliver M., Leparc-Goffart I., Marimoutou C. (2011). Chikungunya Virus Infection. Curr. Infect. Dis. Rep..

[82] Ng L.F., Chow A., Sun Y.J., Kwek D.J., Lim P.L., Dimatatac F., Ng L.C., Ooi E.E., Choo K.H., Her Z. (2009). IL-1beta, IL-6, and RANTES as biomarkers of Chikungunya severity. PLoS ONE.

[83] Kelvin A.A., Banner D., Silvi G., Moro M.L., Spataro N., Gaibani P., Cavrini F., Pierro A., Rossini G., Cameron M.J. (2011). Inflammatory cytokine expression is associated with chikungunya virus resolution and symptom severity. PLoS Neglected Trop. Dis..

[84] Schilte C., Staikovsky F., Couderc T., Madec Y., Carpentier F., Kassab S., Albert M.L., Lecuit M., Michault A. (2013). Chikungunya Virus-associated Long-term Arthralgia: A 36-month Prospective Longitudinal Study. PLoS Neglected Trop. Dis..

[85] Kang H., Auzenbergs M., Clapham H., Maure C., Kim J.-H., Salje H., Taylor C.G., Lim A., Clark A., Edmunds W.J. (2024). Chikungunya seroprevalence, force of infection, and prevalence of chronic disability after infection in endemic and epidemic settings: A systematic review, meta-analysis, and modelling study. Lancet Infect. Dis..

[86] Sissoko D., Malvy D., Ezzedine K., Renault P., Moscetti F., Ledrans M., Pierre V. (2009). Post-epidemic Chikungunya disease on Reunion Island: Course of rheumatic manifestations and associated factors over a 15-month period. PLoS Neglected Trop. Dis..

[87] Chow A., Her Z., Ong E.K., Chen J.M., Dimatatac F., Kwek D.J., Barkham T., Yang H., Rénia L., Leo Y.S. (2011). Persistent arthralgia induced by Chikungunya virus infection is associated with interleukin-6 and granulocyte macrophage colony-stimulating factor. J. Infect. Dis..

[88] Rulli N.E., Rolph M.S., Srikiatkhachorn A., Anantapreecha S., Guglielmotti A., Mahalingam S. (2011). Protection from arthritis and myositis in a mouse model of acute chikungunya virus disease by bindarit, an inhibitor of monocyte chemotactic protein-1 synthesis. J. Infect. Dis..

[89] Chen W., Foo S.S., Taylor A., Lulla A., Merits A., Hueston L., Forwood M.R., Walsh N.C., Sims N.A., Herrero L.J. (2015). Bindarit, an inhibitor of monocyte chemotactic protein synthesis, protects against bone loss induced by chikungunya virus infection. J. Virol..

[90] Morand E.F., Leech M., Bernhagen J. (2006). MIF: A new cytokine link between rheumatoid arthritis and atherosclerosis. Nat. Rev. Drug Discov..

[91] Herrero L.J., Nelson M., Srikiatkhachorn A., Gu R., Anantapreecha S., Fingerle-Rowson G., Bucala R., Morand E., Santos L.L., Mahalingam S. (2011). Critical role for macrophage migration inhibitory factor (MIF) in Ross River virus-induced arthritis and myositis. Proc. Natl. Acad. Sci. USA.

[92] Lin T., Geng T., Harrison A.G., Yang D., Vella A.T., Fikrig E., Wang P. (2020). CXCL10 Signaling Contributes to the Pathogenesis of Arthritogenic Alphaviruses. Viruses.

[93] Morrison T.E., Simmons J.D., Heise M.T. (2008). Complement receptor 3 promotes severe ross river virus-induced disease. J. Virol..

[94] Morrison T.E., Fraser R.J., Smith P.N., Mahalingam S., Heise M.T. (2007). Complement contributes to inflammatory tissue destruction in a mouse model of Ross River virus-induced disease. J. Virol..

[95] Wilson J.A., Prow N.A., Schroder W.A., Ellis J.J., Cumming H.E., Gearing L.J., Poo Y.S., Taylor A., Hertzog P.J., Di Giallonardo F. (2017). RNA-Seq analysis of chikungunya virus infection and identification of granzyme A as a major promoter of arthritic inflammation. PLoS Pathog..

[96] Chen W., Foo S.S., Zaid A., Teng T.S., Herrero L.J., Wolf S., Tharmarajah K., Vu L.D., van Vreden C., Taylor A. (2017). Specific inhibition of NLRP3 in chikungunya disease reveals a role for inflammasomes in alphavirus-induced inflammation. Nat. Microbiol..

[97] de Castro-Jorge L.A., de Carvalho R.V.H., Klein T.M., Hiroki C.H., Lopes A.H., Guimarães R.M., Fumagalli M.J., Floriano V.G., Agostinho M.R., Slhessarenko R.D. (2019). The NLRP3 inflammasome is involved with the pathogenesis of Mayaro virus. PLoS Pathog..

[98] Lidbury B.A., Simeonovic C., Maxwell G.E., Marshall I.D., Hapel A.J. (2000). Macrophage-Induced Muscle Pathology Results in Morbidity and Mortality for Ross River Virus-Infected Mice. J. Infect. Dis..

[99] Lidbury B.A., Rulli N.E., Suhrbier A., Smith P.N., McColl S.R., Cunningham A.L., Tarkowski A., van Rooijen N., Fraser R.J., Mahalingam S. (2008). Macrophage-derived proinflammatory factors contribute to the development of arthritis and myositis after infection with an arthrogenic alphavirus. J. Infect. Dis..

[100] Morrison T.E., Whitmore A.C., Shabman R.S., Lidbury B.A., Mahalingam S., Heise M.T. (2006). Characterization of Ross River virus tropism and virus-induced inflammation in a mouse model of viral arthritis and myositis. J. Virol..

[101] Stoermer K.A., Burrack A., Oko L., Montgomery S.A., Borst L.B., Gill R.G., Morrison T.E. (2012). Genetic ablation of arginase 1 in macrophages and neutrophils enhances clearance of an arthritogenic alphavirus. J. Immunol..

[102] Teo T.H., Lum F.M., Claser C., Lulla V., Lulla A., Merits A., Rénia L., Ng L.F. (2013). A pathogenic role for CD4+ T cells during Chikungunya virus infection in mice. J. Immunol..

[103] Chan Y.H., Teo T.H., Torres-Ruesta A., Hartimath S.V., Chee R.S., Khanapur S., Yong F.F., Ramasamy B., Cheng P., Rajarethinam R. (2020). Longitudinal [18F]FB-IL-2 PET Imaging to Assess the Immunopathogenicity of O’nyong-nyong Virus Infection. Front. Immunol..

[104] Zarrella K.M., Sheridan R.M., Ware B.C., Davenport B.J., da Silva M.O.L., Vyshenska D., Martin A.U., May N.A., Fish E.R., Weiskopf D. (2026). Chikungunya virus persists in joint-associated macrophages and promotes chronic disease in mice. Nat. Microbiol..

[105] Merten E.M., Sears J.D., Leisner T.M., Hardy P.B., Ghoshal A., Hossain M.A., Asressu K.H., Brown P.J., Tse E.G., Stashko M.A. (2024). Identification of a cell-active chikungunya virus nsP2 protease inhibitor using a covalent fragment-based screening approach. Proc. Natl. Acad. Sci. USA.

[106] Weber W.C., Streblow Z.J., Andoh T.F., Denton M., Raué H.-P., Amanna I.J., Slifka D.K., Kreklywich C.N., Arduino I., Sulgey G. (2025). Development of a virulent O’nyong’nyong challenge model to determine heterologous protection mediated by a hydrogen peroxide-inactivated chikungunya virus vaccine. PLoS Neglected Trop. Dis..

[107] Zhang Y., Tang S., Chen L., Song S., Cao J., Tian G., Xiao G., Shen J., Zhang L. (2026). Investigation of a clinical trial drug VV261 as a potent antiviral candidate against Chikungunya virus. Signal Transduct. Target. Ther..

[108] Kovacikova K., van Hemert M.J. (2020). Small-Molecule Inhibitors of Chikungunya Virus: Mechanisms of Action and Antiviral Drug Resistance. Antimicrob. Agents Chemother..

[109] Abdelnabi R., Kovacikova K., Moesslacher J., Donckers K., Battisti V., Leyssen P., Langer T., Puerstinger G., Quérat G., Li C. (2020). Novel Class of Chikungunya Virus Small Molecule Inhibitors That Targets the Viral Capping Machinery. Antimicrob. Agents Chemother..

[110] Kovacikova K., Gorostiola González M., Jones R., Reguera J., Gigante A., Pérez-Pérez M.J., Pürstinger G., Moesslacher J., Langer T., Jeong L.S. (2021). Structural Insights into the Mechanisms of Action of Functionally Distinct Classes of Chikungunya Virus Nonstructural Protein 1 Inhibitors. Antimicrob. Agents Chemother..

[111] Cheng Y., Zheng W., Dong X., Sun T., Xu M., Xiang L., Li J., Wang H., Jian X., Yu J. (2025). Design and Development of a Novel Oral 4′-Fluorouridine Double Prodrug VV261 against SFTSV. J. Med. Chem..

[112] Fox J.M., Long F., Edeling M.A., Lin H., van Duijl-Richter M.K.S., Fong R.H., Kahle K.M., Smit J.M., Jin J., Simmons G. (2015). Broadly Neutralizing Alphavirus Antibodies Bind an Epitope on E2 and Inhibit Entry and Egress. Cell.

[113] Powell L.A., Miller A., Fox J.M., Kose N., Klose T., Kim A.S., Bombardi R., Tennekoon R.N., Dharshan de Silva A., Carnahan R.H. (2020). Human mAbs Broadly Protect against Arthritogenic Alphaviruses by Recognizing Conserved Elements of the Mxra8 Receptor-Binding Site. Cell Host Microbe.

[114] Weber W.C., Streblow Z.J., Kreklywich C.N., Denton M., Sulgey G., Streblow M.M., Marcano D., Flores P.N., Rodriguez-Santiago R.M., Alvarado L.I. (2024). The Approved Live-Attenuated Chikungunya Virus Vaccine (IXCHIQ^®^) Elicits Cross-Neutralizing Antibody Breadth Extending to Multiple Arthritogenic Alphaviruses Similar to the Antibody Breadth Following Natural Infection. Vaccines.

[115] Hwang J., Jiang A., Fikrig E. (2018). Rev-erb Agonist Inhibits Chikungunya and O’nyong’nyong Virus Replication. Open Forum Infect. Dis..

[116] Sandenon Seteyen A.-L., Guiraud P., Gasque P., Girard-Valenciennes E., Sélambarom J. (2023). In Vitro Analyses of the Multifocal Effects of Natural Alkaloids Berberine, Matrine, and Tabersonine against the O’nyong-nyong Arthritogenic Alphavirus Infection and Inflammation. Pharmaceuticals.

[117] Garcia G., Irudayam J.I., Jeyachandran A.V., Dubey S., Chang C., Castillo Cario S., Price N., Arumugam S., Marquez A.L., Shah A. (2023). Innate immune pathway modulator screen identifies STING pathway activation as a strategy to inhibit multiple families of arbo and respiratory viruses. Cell Rep. Med..

[118] Cheng G., Liu Y., Wang P., Xiao X. (2016). Mosquito Defense Strategies against Viral Infection. Trends Parasitol..

[119] Baxter R.H., Steinert S., Chelliah Y., Volohonsky G., Levashina E.A., Deisenhofer J. (2010). A heterodimeric complex of the LRR proteins LRIM1 and APL1C regulates complement-like immunity in *Anopheles gambiae*. Proc. Natl. Acad. Sci. USA.

[120] Povelones M., Upton L.M., Sala K.A., Christophides G.K. (2011). Structure-function analysis of the *Anopheles gambiae* LRIM1/APL1C complex and its interaction with complement C3-like protein TEP1. PLoS Pathog..

[121] Mitri C., Jacques J.-C., Thiery I., Riehle M.M., Xu J., Bischoff E., Morlais I., Nsango S.E., Vernick K.D., Bourgouin C. (2009). Fine Pathogen Discrimination within the APL1 Gene Family Protects *Anopheles gambiae* against Human and Rodent Malaria Species. PLoS Pathog..

[122] Carissimo G., Pondeville E., McFarlane M., Dietrich I., Mitri C., Bischoff E., Antoniewski C., Bourgouin C., Failloux A.B., Kohl A. (2015). Antiviral immunity of *Anopheles gambiae* is highly compartmentalized, with distinct roles for RNA interference and gut microbiota. Proc. Natl. Acad. Sci. USA.

[123] Minaei M.E., Yousefi Nojookambari N., Ghodraty M., Yazdansetad S. (2025). *Wolbachia* as a transformative tool for mosquito-borne disease control: A comprehensive review of mechanisms, efficacy, and future directions. Pathog. Glob. Health.

[124] York A. (2021). Defeating dengue with *Wolbachia*. Nat. Rev. Microbiol..

[125] Rainey S.M., Lefteri D.A., Darby C., Kohl A., Merits A., Sinkins S.P. (2024). Evidence of Differences in Cellular Regulation of *Wolbachia*-Mediated Viral Inhibition between Alphaviruses and Flaviviruses. Viruses.

[126] Waldock J., Olson K.E., Christophides G.K. (2012). *Anopheles gambiae* antiviral immune response to systemic O’nyong-nyong infection. PLoS Neglected Trop. Dis..

[127] Keene K.M., Foy B.D., Sanchez-Vargas I., Beaty B.J., Blair C.D., Olson K.E. (2004). RNA interference acts as a natural antiviral response to O’nyong-nyong virus (Alphavirus; Togaviridae) infection of *Anopheles gambiae*. Proc. Natl. Acad. Sci. USA.

[128] Olson K.E., Blair C.D. (2015). Arbovirus-mosquito interactions: RNAi pathway. Curr. Opin. Virol..

[129] Sim C., Hong Y.S., Tsetsarkin K.A., Vanlandingham D.L., Higgs S., Collins F.H. (2007). *Anopheles gambiae* heat shock protein cognate 70B impedes o’nyong-nyong virus replication. BMC Genom..

[130] Qu J., Schinkel M., Chiggiato L., Rosendo Machado S., Overheul G.J., Miesen P., van Rij R.P. (2025). The Hsf1-sHsp cascade has pan-antiviral activity in mosquito cells. Commun. Biol..

[131] da Silva A.F., Machado L.C., de Paula M.B., da Silva Pessoa Vieira C.J., de Morais Bronzoni R.V., de Melo Santos M.A.V., Wallau G.L. (2020). Culicidae evolutionary history focusing on the Culicinae subfamily based on mitochondrial phylogenomics. Sci. Rep..

[132] Vanlandingham D.L., Hong C., Klingler K., Tsetsarkin K., McElroy K.L., Powers A.M., Lehane M.J., Higgs S. (2005). Differential infectivities of o’nyong-nyong and chikungunya virus isolates in *Anopheles gambiae* and *Aedes aegypti* mosquitoes. Am. J. Trop. Med. Hyg..

[133] Vanlandingham D.L., Tsetsarkin K., Klingler K.A., Hong C., McElroy K.L., Lehane M.J., Higgs S. (2006). Determinants of vector specificity of o’nyong nyong and chikungunya viruses in *Anopheles* and *Aedes* mosquitoes. Am. J. Trop. Med. Hyg..

[134] Saxton-Shaw K.D., Ledermann J.P., Borland E.M., Stovall J.L., Mossel E.C., Singh A.J., Wilusz J., Powers A.M. (2013). O’nyong nyong Virus Molecular Determinants of Unique Vector Specificity Reside in Non-Structural Protein 3. PLoS Neglected Trop. Dis..

[135] Byers N.M., Burns P.L., Stuchlik O., Reed M.S., Ledermann J.P., Pohl J., Powers A.M. (2023). Identification of mosquito proteins that differentially interact with alphavirus nonstructural protein 3, a determinant of vector specificity. PLoS Neglected Trop. Dis..

[136] Eckei L., Krieg S., Bütepage M., Lehmann A., Gross A., Lippok B., Grimm A.R., Kümmerer B.M., Rossetti G., Lüscher B. (2017). The conserved macrodomains of the non-structural proteins of Chikungunya virus and other pathogenic positive strand RNA viruses function as mono-ADP-ribosylhydrolases. Sci. Rep..

[137] McPherson R.L., Abraham R., Sreekumar E., Ong S.-E., Cheng S.-J., Baxter V.K., Kistemaker H.A.V., Filippov D.V., Griffin D.E., Leung A.K.L. (2017). ADP-ribosylhydrolase activity of Chikungunya virus macrodomain is critical for virus replication and virulence. Proc. Natl. Acad. Sci. USA.

[138] Götte B., Liu L., McInerney G.M. (2018). The Enigmatic Alphavirus Non-Structural Protein 3 (nsP3) Revealing Its Secrets at Last. Viruses.

[139] Lark T., Keck F., Narayanan A. (2018). Interactions of Alphavirus nsP3 Protein with Host Proteins. Front. Microbiol..

[140] Gao Y., Goonawardane N., Ward J., Tuplin A., Harris M. (2019). Multiple roles of the non-structural protein 3 (nsP3) alphavirus unique domain (AUD) during Chikungunya virus genome replication and transcription. PLoS Pathog..

[141] Alhammad Y.M.O., Fehr A.R. (2020). The Viral Macrodomain Counters Host Antiviral ADP-Ribosylation. Viruses.

[142] Abdullah N., Ahemad N., Aliazis K., Khairat J.E., Lee T.C., Abdul Ahmad S.A., Adnan N.A.A., Macha N.O., Hassan S.S. (2021). The Putative Roles and Functions of Indel, Repetition and Duplication Events in Alphavirus Non-Structural Protein 3 Hypervariable Domain (nsP3 HVD) in Evolution, Viability and Re-Emergence. Viruses.

[143] Jayabalan A.K., Adivarahan S., Koppula A., Abraham R., Batish M., Zenklusen D., Griffin D.E., Leung A.K.L. (2021). Stress granule formation, disassembly, and composition are regulated by alphavirus ADP-ribosylhydrolase activity. Proc. Natl. Acad. Sci. USA.

[144] Teppor M., Žusinaite E., Merits A. (2021). Phosphorylation Sites in the Hypervariable Domain in Chikungunya Virus nsP3 Are Crucial for Viral Replication. J. Virol..

[145] Strauss E.G., Rice C.M., Strauss J.H. (1983). Sequence coding for the alphavirus nonstructural proteins is interrupted by an opal termination codon. Proc. Natl. Acad. Sci. USA.

[146] Myles K.M., Kelly C.L., Ledermann J.P., Powers A.M. (2006). Effects of an opal termination codon preceding the nsP4 gene sequence in the O’Nyong-Nyong virus genome on *Anopheles gambiae* infectivity. J. Virol..

[147] Mameli E., Samantsidis G.-R., Viswanatha R., Kwon H., Hall D.R., Butnaru M., Hu Y., Mohr S.E., Perrimon N., Smith R.C. (2025). A genome-wide CRISPR screen in *Anopheles* mosquito cells identifies fitness and immune cell function-related genes. Nat. Commun..

[148] Pabst R., Sousa C.A., Essl F., García-Rodríguez A., Liu D., Lenzner B., Schertler A., Zêzere J.L., Capinha C. (2025). Global invasion patterns and dynamics of disease vector mosquitoes. Nat. Commun..

[149] Tappe D., Kapaun A., Emmerich P., Campos Rde M., Cadar D., Günther S., Schmidt-Chanasit J. (2014). O’nyong-nyong virus infection imported to Europe from Kenya by a traveler. Emerg. Infect. Dis..

[150] Faria N.R., Azevedo R., Kraemer M.U.G., Souza R., Cunha M.S., Hill S.C., Thézé J., Bonsall M.B., Bowden T.A., Rissanen I. (2016). Zika virus in the Americas: Early epidemiological and genetic findings. Science.

[151] Musso D., Gubler D.J. (2016). Zika Virus. Clin. Microbiol. Rev..

[152] Ahmed A., Khogali R., Elnour M.-A.B., Nakao R., Salim B. (2021). Emergence of the invasive malaria vector *Anopheles stephensi* in Khartoum State, Central Sudan. Parasit. Vectors.

[153] Tadesse F.G., Ashine T., Teka H., Esayas E., Messenger L.A., Chali W., Meerstein-Kessel L., Walker T., Wolde Behaksra S., Lanke K. (2021). *Anopheles stephensi* Mosquitoes as Vectors of Plasmodium vivax and falciparum, Horn of Africa, 2019. Emerg. Infect. Dis..

[154] Mutsaers M., Engdahl C.S., Wilkman L., Ahlm C., Evander M., Lwande O.W. (2023). Vector competence of *Anopheles stephensi* for O’nyong-nyong virus: A risk for global virus spread. Parasit. Vectors.

[155] della Torre A., Caputo B., De Marco C.M., Perugini E., Pombi M. (2026). *Anopheles* *coluzzii*. Trends Parasitol..

[156] Carissimo G., Pain A., Belda E., Vernick K.D. (2018). Highly focused transcriptional response of *Anopheles coluzzii* to O’nyong nyong arbovirus during the primary midgut infection. BMC Genom..

[157] Terradas G., Novelo M., Metz H., Brustolin M., Rasgon J.L. (2023). *Anopheles albimanus* is a Potential Alphavirus Vector in the Americas. Am. J. Trop. Med. Hyg..

[158] Jagtap S., Altinli M., Badusche M., Chevalier M., Becker N., Leggewie M., Schnettler E. (2025). Invasive *Aedes albopictus* is a competent vector for O’nyong Nyong virus. One Health.

